# The caspase-6–p62 axis modulates p62 droplets based autophagy in a dominant-negative manner

**DOI:** 10.1038/s41418-021-00912-x

**Published:** 2021-12-03

**Authors:** Evelina Valionyte, Yi Yang, Sophie A. Griffiths, Amelia T. Bone, Elizabeth R. Barrow, Vikram Sharma, Boxun Lu, Shouqing Luo

**Affiliations:** 1 Peninsula Medical School, Faculty of Health, University of Plymouth, Research Way, Plymouth, PL6 8BU UK; 2 School of Biomedical Sciences, Faculty of Health, University of Plymouth, Drake Circus, Plymouth, PL4 8AA UK; 3 State Key Laboratory of Medical Neurobiology, School of Life Sciences, Fudan University, Shanghai, 200438 China

**Keywords:** Macroautophagy, Proteases

## Abstract

SQSTM1/p62, as a major autophagy receptor, forms droplets that are critical for cargo recognition, nucleation, and clearance. p62 droplets also function as liquid assembly platforms to allow the formation of autophagosomes at their surfaces. It is unknown how p62-droplet formation is regulated under physiological or pathological conditions. Here, we report that p62-droplet formation is selectively blocked by inflammatory toxicity, which induces cleavage of p62 by caspase-6 at a novel cleavage site D256, a conserved site across human, mouse, rat, and zebrafish. The N-terminal cleavage product is relatively stable, whereas the C-terminal product appears undetectable. Using a variety of cellular models, we show that the p62 N-terminal caspase-6 cleavage product (p62-N) plays a dominant-negative role to block p62-droplet formation. In vitro p62 phase separation assays confirm this observation. Dominant-negative regulation of p62-droplet formation by caspase-6 cleavage attenuates p62 droplets dependent autophagosome formation. Our study suggests a novel pathway to modulate autophagy through the caspase-6–p62 axis under certain stress stimuli.

## Introduction

Macroautophagy (referred to as autophagy) is a lysosome-dependent degradation pathway for the clearance of aberrant cytoplasmic constituents. The process includes autophagosome formation and maturation, autophagosome–lysosome fusion, and cargo degradation [1]. Upon certain stress signals, autophagosome formation begins from a phagophore in the cytoplasm. During autophagosome elongation, ATG8/LC3 family proteins are conjugated to the membrane lipid phosphatidylethanolamine (PE) by ATG7 and ATG3-mediated ubiquitination-like mechanisms with stimulation by the ATG5-12/ATG16L1 complex, and become associated with the autophagosome membrane at the leading edge of a phagophore, where packing defects in the highly curved membranes allow the local enrichment of PE, leading to LC3 localization to high-curvature regions [2–4]. As the autophagosome elongates, cytoplasmic contents or cargo materials are gradually encapsulated and isolated from the rest of the cytoplasm. Nascent autophagosomes undergo maturation by fusing with endolysosomal vesicles to form acidified amphisomes, which further fuse with lysosomes to become digestive autolysosomes [5].

Receptors mediated autophagy is defined as selective autophagy, which selectively removes cargo materials, for instance, misfolded protein aggregates (aggrephagy) [6–11], intracellular pathogens (xenophagy) [12–14] and damaged mitochondria (mitophagy) [15–19]. During selective autophagy, cargos may drive the local assembly of autophagosomes keeping the membrane close to the cargo for its engulfment [20–24]. As a major autophagy receptor, p62 harbors a Phox and Bem1p (PB1) domain, an LC3-interacting region (LIR) motif and a ubiquitin-associated (UBA) domain [25–27]. p62 recruits the polyubiquitinated cargo through its C-terminal UBA domain, and its binding to LC3 enables the recruited cargo to be selectively enclosed by the autophagosome. The PB1 domain at the N-terminus mediates p62 self-oligomerization [27], which is critical for the role of p62 in cargo recognition and LC3 interaction. p62, cooperating with other protein factors including WDR81, ALFY or huntingtin [28–31], mediates the nucleation and clearance of protein aggregates [6–8, 32–35].

p62 filaments/bodies are formed via protein liquid–liquid phase separation [36, 37], a process in which biomolecules demix from solution to form protein droplets [38]. p62-droplet formation requires polyubiquitinated protein binding at its UBA domain [36, 39]. Recently, Kageyama et al. provided compelling evidence showing that p62 droplets function as platforms for the formation of the autophagosome [40], and Agudo-Canalejo et al. [41] described that autophagosomes initiate from phase-separated droplets through p62-droplet surface tension, and wetting regulates autophagosomal sheet bending for autophagosome completion, resulting in droplet autophagy or cytosolic autophagy. The p62 droplet involves the formation of flattened autophagic membrane sheets that contact the surface of the droplet. With the surface tension of the p62 droplet below or beyond its critical point, droplets based autophagosome synthesis can occur through the modes of piecemeal sequestration or complete sequestration. The droplet size influences the critical surface tension; too large or too small droplet size can lead to the failure of piecemeal sequestration or complete sequestration, respectively, due to membrane supply limitation. The appropriate size of p62 droplets would thus be crucial for autophagosomal formation.

p62 signaling is regulated at both the transcriptional and the posttranslational level. NF-κB controls p62 gene transcription [42]. p62 expression is upregulated by a variety of stressors such as oxidative stress, protein aggregation and proinflammatory cytokines, which promote the activation of NF-κB pathway [43–45]. p62 phosphorylation and ubiquitination modify its oligomerization and cargo recognition [46]. We have recently found that DAXX positively regulates p62-droplet formation and protects against reactive oxidative toxicity [37, 47]. However, it is poorly understood how p62-droplet formation is regulated under physiological or pathological conditions. Here, we show that p62-droplet formation was selectively blocked by inflammatory toxicity. Mechanistically, p62 was cleaved by caspase-6 at D256 in inflammatory stimuli. The N-terminal fragment of p62 plays a dominant-negative role in regulating p62-droplet formation and p62 droplets based autophagy. This suggests that the caspase-6–p62 axis serves as a novel pathway to modulate autophagy, potentially involved in inflammation-associated toxicity in relevant pathological conditions.

## Results

### Cytotoxicity-modulated p62-droplet formation does not correspond to its protein levels

**Fig. 1:**
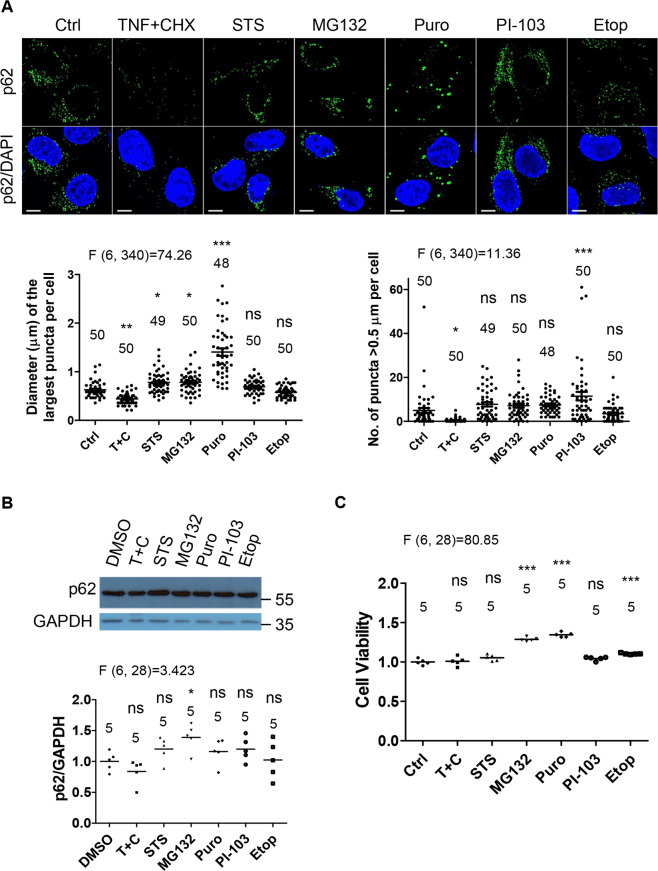
Cytotoxicity-modulated p62-droplet formation does not correspond to full-length p62 levels. **A–C** p62-droplet formation in cells treated with an array of cytotoxicity. HeLa cells were treated with vehicle (DMSO) for 4 h, TNFα (10 ng/ml) + CHX (50 µg/ml) (T + C) for 3 h, staurosporine (STS) (1 µM), MG132 (5 µM), puromycin (Puro) (5 µg/ml), PI-103 (1 µM) or Etoposide (Etop) (50 µM) for 4 h, respectively. **A** The cells were stained with anti-p62 antibody (produced in guinea pig). Confocal images were acquired with confocal microscopy. Bar: 10 µm. The diameter of the biggest p62 puncta (µm) in each cell was measured (ImageJ), and the number of p62 puncta > 0.5 µm in each cell was assessed (ImageJ). *n* = the number of cells, as shown in each plot. **B** Full-length p62 levels in the cells under cytotoxicity. The cells were lysed and subjected to immunoblot with anti-p62 and GAPDH antibodies, successively. Data were quantified with ImageJ. *n* = independent immunoblots. **C** Cell viability under the array of cytotoxicity. The cells were subjected to viability assays with the CellTiter-Glo Luminescent Cell Viability Assay kit (Promega). *n* = independently plated wells. Data are shown as mean ± sem (**A**). Statistical analysis was performed by one-way ANOVA with Dunnett’s multiple comparison test. The F/degree of freedom/post hoc *P* values is indicated in each plot. ns not significant; *P < 0.05; **P < 0.01; ***P < 0.0001.

To understand how p62 droplets based autophagy is dynamically regulated under stress conditions, we initially investigated p62-droplet formation in response to a variety of toxic agents, namely staurosporine, a potent protein kinase inhibitor to induce cytotoxicity [48]. MG132, a tripeptide aldehyde inhibitor of 26S proteasome that induces accumulation of polyubiquitinated misfolded proteins [49, 50]; puromycin, a proteotoxin that induces premature release of polypeptide chains from ribosomes [51]; PI-103, a kinase inhibitor for PI-3 kinase/Akt and mTOR exerting cell death [52] or etoposide, a DNA topoisomerase II inhibitor causing genotoxic stress [53], in addition to TNFα stimulus. We observed that p62 droplet levels were subject to an increase or a decrease according to treatment settings (Fig. 1A). Of note, TNFα induces cytotoxicity signaling and NF-κB activation [54, 55], and cycloheximide (CHX) was used to block NF-κB survival signal. Interestingly, the droplet formation did not correspond to p62 protein levels in these conditions (Fig. 1B). This suggests that other factors, in addition to p62 protein levels, would contribute to p62-droplet formation. We did not observe occurrence of cell death in the treatment conditions (Fig. 1C), suggesting that cell death did not affect p62-droplet formation in these cases. Among these toxic stimuli, p62-droplet formation was significantly weakened only in TNFα + CHX treatment (Fig. 1A). Both MG132 and puromycin significantly increased p62 puncta size, presumably because they enhances protein ubiquitination levels in cells; staurosporine significantly increased p62 puncta size, and PI-103 did not alter the size of p62 puncta, but increased the number of p62 puncta, while etoposide did not significantly change either the number or the size of p62 puncta. We confirmed that inhibition of NF-κB pathway was critical to potentiate the effect of TNFα on p62-droplet formation. p62-droplet formation was markedly weakened under the condition (Fig. S1A), although the full-length p62 level was not significantly affected in the cells treated with TNFα + IKK-16 (inhibitor of ΙκΒ kinase (IKK) to block NF-κB signal) (Fig. S1B).

### Reduction in p62-droplet formation is associated with a novel p62 species

**Fig. 2:**
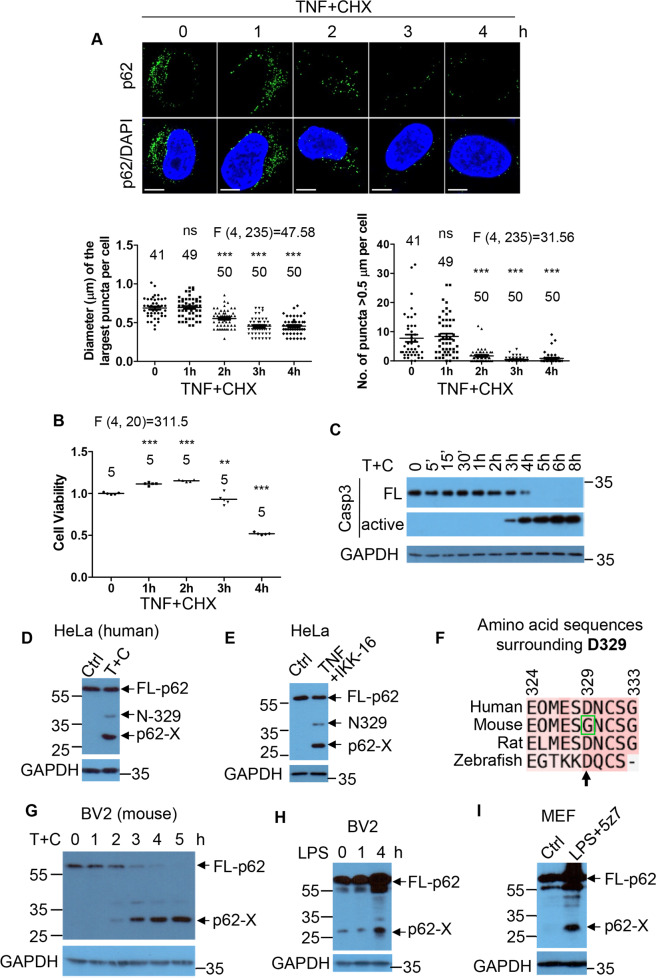
Attenuation in p62-droplet formation is associated with a novel p62 species. **A**, **B** HeLa cells were treated with TNFα (10 ng/ml) + CHX (50 µg/ml) (T + C) for 0, 1, 2, 3 or 4 h, respectively. **A** p62-droplet formation in the cells under a time-course TNFα toxicity. The cells were stained with guinea pig anti-p62 antibody. Confocal images were acquired with confocal microscopy. Bar: 10 µm. The diameter of the biggest p62 puncta (µm) in each cell, and the number of p62 puncta > 0.5 µm in each cell were assessed by ImageJ. *n* = the number of cells, as shown in each plot. **B** Cell viability under a time-course TNFα toxicity. The cells were subjected to viability assays with the CellTiter-Glo Luminescent Cell Viability Assay kit (Promega) *n* = independently plated wells, as shown in each plot. Data are shown as mean ± sem (**A**). Statistical analysis was performed by one-way ANOVA with Dunnett’s multiple comparison test. The F/degree of freedom/post hoc *P* values is indicated in each plot. ns not significant; **P < 0.01; ***P < 0.0001. **C** Caspase-3 activation in cells under a time-course TNFα toxicity. HeLa cells were treated with TNFα (10 ng/ml) + CHX (50 µg/ml) (T + C) for 0, 5 min, 15 min, 30 min, 1, 2, 3, 4, 5, 6 or 8 h, respectively. The cells were lysed and subjected to immunoblot with anti-caspase-3 and GAPDH antibodies, successively. **D** p62 was subjected to a potential novel major cleavage under TNFα toxicity. HeLa cells were treated with the vehicle (DMSO) (Ctrl) or TNFα (10 ng/ml) + CHX (50 µg/ml) (T + C) for 4 h. The cell lysates were subjected to immunoblot with anti-p62 (N-terminus) and GAPDH successively. N-329: p62 N-terminus 1-329aa. p62-X: a major short p62 isoform under investigation. **E** HeLa cells were treated with the vehicle (DMSO) or TNFα (10 ng/ml) + IKK-16 (1 µM) for 4 h, and the cell lysates were subjected to anti-p62 (N-terminus) and GAPDH successively. **F** Alignment of p62 from the indicated species, showing that G329 is not a consensus caspase cleavage site in mouse p62. **G** p62 was subjected to a single band conversion under a time-course TNFα toxicity. Mouse microglia BV2 cells were treated with mouse TNFα (10 ng/ml) + CHX (50 µg/ml) (T + C) for 0, 1, 2, 3, 4 or 5 h, respectively. The cells were lysed and subjected to immunoblot with anti-p62 (N-terminus) and GAPDH successively. **H** Mouse BV2 cells were treated with LPS for 0, 1, or 4 h. The cells were lysed and subjected to immunoblot with anti-p62 (N-terminus) and GAPDH successively. **I** MEFs were treated with the vehicle (DMSO) or LPS (1 µg/ml) + 5z7 (0.5 µM), to mimic Yersinia infection for TAK1 inhibition, for 4 h, and the cell lysates were subjected to anti-p62 (N-terminus) and GAPDH successively.

We aimed to characterize the effect of TNFα treatment on p62-droplet formation, and found that p62-droplet formation began to reduce at 2-h post TNFα + CHX treatment (Fig. 2A), when cell death did not occur yet (Fig. 2B), and the executioner caspase-3 did not undergo activation at 2-h TNFα + CHX treatment (Fig. 2C). We observed a major shorter isoform of p62 (p62-X) in the HeLa cells treated with TNFα + CHX (Fig. 2D). Notably, p62-X, detected by an antibody against p62 N-terminus, was migrated with a size of ~30 kDa, predictably from a cleavage between amino acid residue (aa) 200 and 300. Caspase-1 [56] and casaspe-8 [57] were suggested to cleave p62 at aspartic acid 329 (D329). Interestingly, D329 cleavage by caspase-1/8 appeared to be relatively minor in our conditions (Fig. 2D). Similarly, TNFα treatment along with IKK-16 inhibition of the NF-κB pathway induced p62-X production (Fig. 2E). D329 in human p62 is not conserved in the mouse counterpart (Fig. 2F), and G329 in mouse p62 is predicted not to be cleaved by a caspase. However, p62-X was produced in BV2 mouse microglial cells treated with TNFα + CHX (Fig. 2G). This suggests that a new cleavage of p62 could occur under TNFα + CHX treatment. Such potential p62 cleavage occurred in human and mouse cells under TNFα, or lipopolysaccharide (LPS) toxicity (Fig. 2H, I, Fig. S2A, B), implying a potential role of the new p62 species in p62-droplet formation under inflammatory stress conditions. TNFα alone also induced the new p62 species formation in COLO-205 cells, although the effect was milder than that with NF-κB inhibition (Fig. S2C, D).

### p62 cleavage occurs at D256

**Fig. 3:**
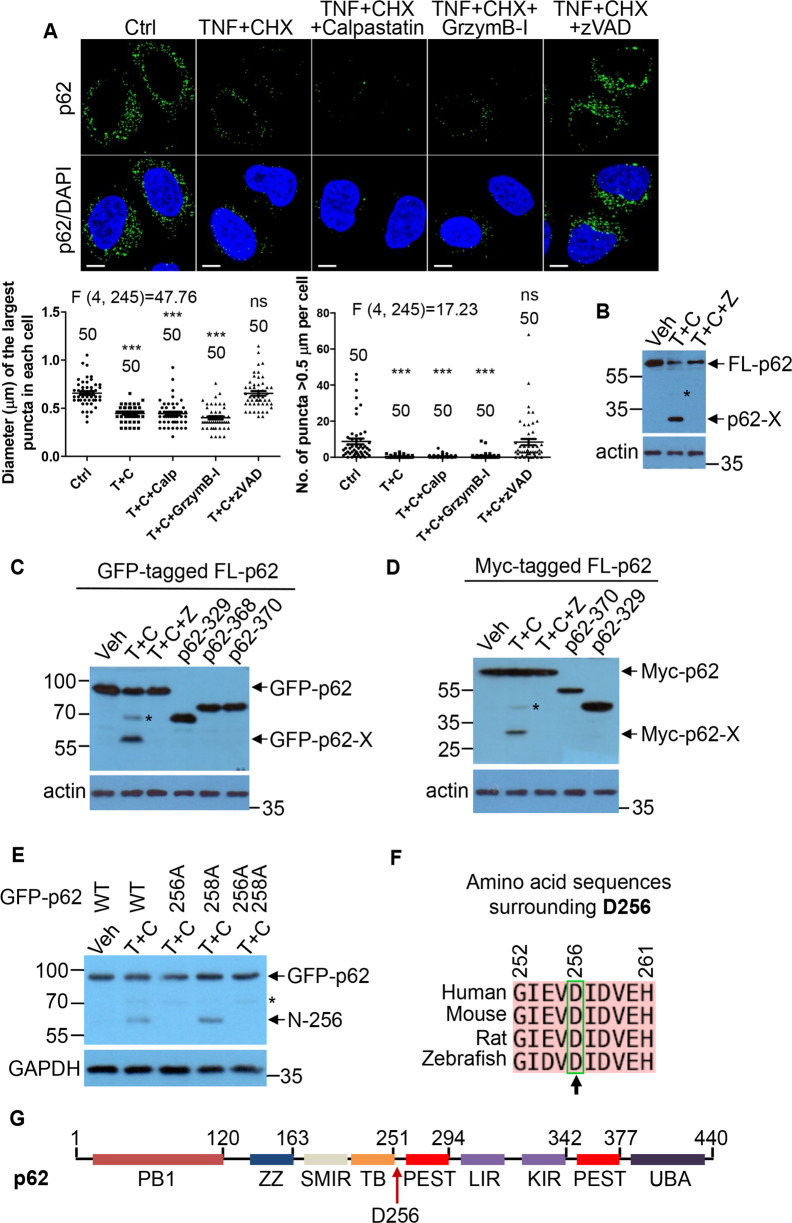
p62 is subjected to a novel caspase cleavage at D256. **A** The pan-caspase inhibitor restored p62-droplet formation. HeLa cells were treated with vehicle (DMSO), TNFα (10 ng/ml) + CHX (50 µg/ml), or TNFα (10 ng/ml) + CHX (50 µg/ml) along with the calpain inhibitor calpastatin (Calp) (20 µM), granzyme B inhibitor (GrzymB-I) (20 µM), or the pan-caspase inhibitor z-VAD-fmk (z-VAD) (20 µM) for 3 h. The cells were stained with anti-p62 antibody (guinea pig). Confocal images were acquired with confocal microscopy. Bar: 10 µm. The diameter of the biggest p62 puncta (µm) in each cell, and the number of p62 puncta > 0.5 µm in each cell were assessed by ImageJ. *n* = the number of cells, as shown in each plot. Data are shown as mean ± sem. Statistical analysis was performed by one-way ANOVA with Dunnett’s multiple comparison test. The F/degree of freedom/post hoc *P* values are indicated in each plot. ns not significant; ***P < 0.0001. **B** The pan-caspase inhibitor blocked p62 cleavage. HeLa cells were treated with the vehicle (DMSO) (Ctrl), TNFα (10 ng/ml) + CHX (50 µg/ml) (T + C), or TNFα (10 ng/ml) + CHX (50 µg/ml) + z-VAD-fmk (20 µM) (T + C + Z), for 4 h. The cell lysates were subjected to immunoblot with anti-p62 (N-terminus) and β-actin antibody, successively. An asterisk symbol An asterisk symbol (*) denotes p62-1-329aa cleavage fragment. p62-X: the major short p62 isoform under investigation. **C** Mapping the p62 cleavage site using GFP-tagged p62. HeLa cells were transfected with GFP-p62 (lanes 1–3), GFP-p62-1-329aa, GFP-p62-1-368aa or GFP-p62-1-370aa. After 20 h, the cells were treated with vehicle (lane 1), TNFα (10 ng/ml) + CHX (50 µg/ml) (T + C) or TNFα (10 ng/ml) + CHX (50 µg/ml) + z-VAD-fmk (20 µM) (T + C + Z) for 4 h. The cell lysates were subjected to western blot with anti-GFP or β-actin antibody, respectively. An asterisk symbol (*) denotes GFP-p62-1-329aa cleavage fragment. **D** Mapping the p62 cleavage site using Myc-tagged p62. HeLa cells were transfected with Myc-p62 (lanes 1–3), GFP-p62-1-370aa, or Myc-p62-1-329aa. After 20 h, the cells were treated with vehicle (lane 1), TNFα (10 ng/ml) + CHX (50 µg/ml) (lane 2) or TNFα (10 ng/ml) + CHX (50 µg/ml) + z-VAD-fmk (20 µM) (lane 3) for 4 h. The cell lysates were subjected to western blot with anti-Myc or β-actin antibody, respectively. An asterisk symbol (*) denotes Myc-p62-1-329aa cleavage fragment. **E** p62 cleavage occurred at D256. HeLa cells expressing GFP-p62, GFP-p62D256A, GFP-p62D258A, or GFP-p62D256A D258A were treated with TNFα + CHX for 4 h. The cell lysates were subjected to immunoblot with anti-GFP or GAPDH, respectively. An asterisk symbol (*) denotes GFP-p62-1-329aa cleavage fragment. **F** Alignment of p62 from the indicated species, showing D256 is a consensus caspase cleavage site. **G** The diagram of p62 domains showing the cleavage site D256 locates between the TRAF6-binding (TB) domain and the PEST domain.

We used an array of protease inhibitors to reverse the reduction of p62-droplet formation by TNFα toxicity. The pan-caspase inhibitor z-VAD-fmk (z-VAD), but not the calpain inhibitor calpastatin and granzyme B inhibitor, could restore p62-droplet formation (Fig. 3A). These suggest that caspase activity may be critical to reduce p62-droplet formation under TNFα toxicity. Indeed, z-VAD readily blocked the potential new p62 cleavage caused by TNFα + CHX treatment (Fig. 3B), suggesting that caspases could mediate a cleavage for the production of the new major p62 isoform. The full-length p62 level did not significantly reduce at 3-h TNFα treatment (Fig. 1B), while p62-droplet formation was significantly affected under the conditions (Figs. 1A and 2A). We thus hypothesized that p62 cleavage fragments could play a role in the reduction of p62-droplet formation.

To identify the novel cleavage site, N-terminally GFP or Myc-tagged p62 fragments were used as a size reference for the fragment of p62 cleavage induced by TNFα + CHX (Fig. 3C, D). We located the major p62 cleavage site among amino acid residues (aa) 250-300, according to the apparent size of the N-terminal fragment. This allowed us to define aspartic acid 256 (D256) as the cleavage site, as the mutation of D256-to-alanine (A) completely blocked the cleavage caused by TNFα + CHX (Fig. 3E). Note that the D256-to-glutamate (E) mutation created an artificial D258 cleavage site due to the amino acid sequence change after mutagenesis (data not shown). Therefore, p62 D256A, instead of p62 D256E, was used as a noncleavable p62 mutant. In contrast with D329 that is not conserved in mouse, D256 is conserved across the species including human, mouse, rat and zebrafish (Fig. 3F). D256 is localized at the disordered region between the TB domain and PEST domain (Fig. 3G). We excluded the possibility that p62-N could potentially cause cytotoxicity, as we did not observe any toxic effect caused by p62-N (Fig. S3). Collectively, D256 was the major cleavage site in human p62, and the only one in mouse p62 under the inflammatory toxicity conditions.

### Caspase-6 mediates p62 cleavage at D256

**Fig. 4:**
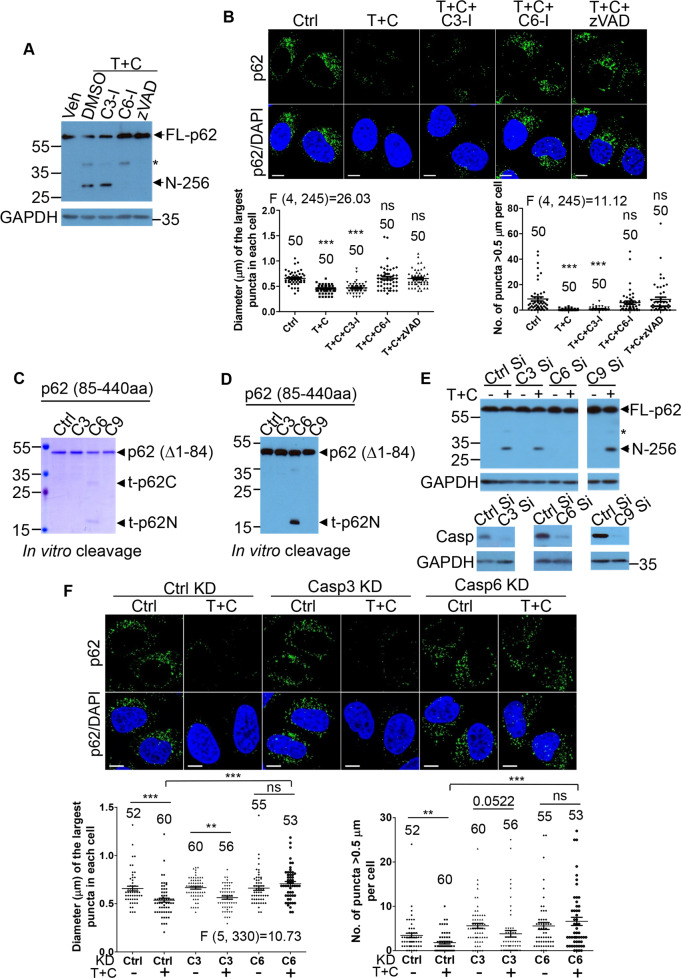
Caspase-6 mediates p62 cleavage at D256. **A** Caspase-6 inhibitor blocked p62 cleavage at D256. HeLa cells were treated with vehicle control, or TNFα (10 ng/ml) + CHX (50 µg/ml) along with DMSO, caspase-3 inhibitor (C3-I) (20 µM), caspase-6 inhibitor (C6-I) (20 µM), or z-VAD-fmk (z-VAD) (20 µM) for 4 h. Cell lysates were subjected to immunoblot with anti-p62 or GAPDH antibodies successively. An asterisk symbol (*) denotes p62-1-329aa cleavage fragment. N-256: p62 N-terminus 1-256aa. **B** Caspase-6 inhibitor or pan-caspase inhibitor restored p62-droplet formation. HeLa cells were treated with vehicle (DMSO), TNFα (10 ng/ml) + CHX (50 µg/ml) (T + C), and TNFα (10 ng/ml) + CHX (50 µg/ml) along with DMSO, caspase-3 inhibitor (C3-I) (20 µM), caspase-6 inhibitor (C6-I) (20 µM) or z-VAD-fmk (z-VAD) (20 µM), for 3 h. The cells were stained with anti-p62 antibody (guinea pig). Confocal images were acquired with confocal microscopy. Bar: 10 µm. The diameter of the biggest p62 puncta (µm) in each cell was measured, and the number of p62 puncta > 0.5 µm in each cell was assessed (ImageJ). *n* = the number of cells, as shown in each plot. Data are shown as mean ± sem. Statistical analysis was performed by one-way ANOVA with Dunnett’s multiple comparison test. The F/degree of freedom/post hoc *P* values are indicated in each plot. ns not significant; ***P < 0.0001. **C**, **D** In vitro cleavage of p62 by caspase-6. For caspase-6 in vitro cleavage assay, recombinant 6× His-tagged p62 (del 1-84aa) was cleaved by control (buffer only) (Ctrl), caspase-3 (C3), caspase-6 (C6) or caspase-9 (C9) in vitro. The reaction mixture was resolved by SDS-PAGE (the arrows mark N- or C-terminal p62), and subjected to Coomassie blue staining (**C**) or subjected to immunoblot with anti-p62 (N-terminus) (**D**). Note that the mobility shift of the p62 N-terminal fragment here differs from those in other occasions of p62 N-terminus, as p62 del 1-84aa was used here. **E** Caspase-6 knockdown blocked p62 cleavage. HeLa cells were knocked down with siRNA for control (Ctrl), caspase-3 (C3), caspase-6 (C6), or caspase-9 (C9) in duplicate, respectively. After 48 h, cells were treated with DMSO (-) or TNFα (10 ng/ml) + CHX (50 µg/ml) (+), as indicated, for 4 h. Cell lysates were subjected to immunoblot with anti-p62 (N-terminus) or GAPDH successively. In parallel (lower panels), samples for control knockdown, caspase-3 knockdown, caspase-6 knockdown, or caspase-9 knockdown were probed with anti-caspase-3 (left), anti-caspase-6 (middle) or anti-caspase-9 antibody (right) to validate knockdown effectiveness. An asterisk symbol (*) denotes p62-1-329aa cleavage fragment. N-256: p62 N-terminus 1-256aa. **F** Caspase-6 knockdown restored p62-droplet formation. HeLa cells were knocked down (KD) with siRNA for control (Ctrl), caspase-3 (C3), or caspase-6 (C6) in duplicate, respectively. After 48 h, cells were treated with DMSO or TNFα (10 ng/ml) + CHX (50 µg/ml) (T + C), as indicated, for 3 h. The cells were stained with anti-p62 antibody (guinea pig). Confocal images were acquired with confocal microscopy. Bar: 10 µm. The diameter of the biggest p62 puncta (µm) in each cell, and the number of p62 puncta > 0.5 µm in each cell were assessed by ImageJ. *n* = the number of cells, as shown in each plot. Data are shown as mean ± sem. Left panel: Statistical analysis was performed by one-way ANOVA with Bonferroni multiple comparison test. The F/degree of freedom/post hoc *P* values are indicated in each plot. ns not significant; **P < 0.01; ***P < 0.0001. Right panel: Statistical analysis was performed by unpaired/two-tailed T-test. ns not significant; **P = 0.006; ***P < 0.0001.

z-VAD could restore the inhibitory effect on p62-droplet formation, and blocked p62 cleavage (Fig. 3A, B), suggesting that caspases are involved in p62 cleavage at D256. We investigated which caspases mediated the cleavage. We found that caspase-6 inhibitor, but not caspase-3 inhibitor, reduced the cleavage of p62 (Fig. 4A). We tested if inhibition of caspase-6 activity could restore p62-droplet reduction by TNFα toxicity. z-VAD and the caspase-6 inhibitor, which reversed caspase-6-mediated p62 cleavage, restored p62-droplet formation in the cells treated with TNFα + CHX. By contrast, the caspase-3 inhibitor was unable to recover p62-droplet formation in the conditions (Fig. 4B). The treatment of LPS/TNFα and 5Z-7-oxozeaenol (5z7), the inhibitor of TAK1, is used to mimic the effect of Yersinia pathogen infection [58, 59], since Yersinia species bacteria rely on the effector protein YopJ to block activation of TAK1 and NF-κB pathway toward cell death and inflammation [60, 61]. TNFα treatment with TAK1 inhibition by 5z7 caused substantial p62 cleavage at D256 (Fig. S4A) and a significant reduction in p62-droplet formation in COLO-205 cells (Fig. S4B). Caspase-6 inhibition or pan-caspase inhibition by z-VAD-fmk effectively reversed TNFα + 5z7-caused p62 cleavage and reduced droplet formation in these cells (Fig. S4A, B). In vitro assays confirmed that caspase-6 directly cleaved p62, but caspase-3 and caspase-9 did not, as shown in both Coomassie blue staining (Fig. 4C) and immunoblot (Fig. 4D). Caspase-6 knockdown blocked p62 cleavage at D256 in response to TNFα + CHX treatment (see lane 6), while caspase-3 or caspase-9 knockdown did not reduce such p62 cleavage by TNFα + CHX treatment (Fig. 4E). Furthermore, caspase-6 knockdown rather than caspase-3 knockdown significantly increased p62-droplet formation in the droplet size and number (Fig. 4F). Collectively, these data demonstrated that caspase-6 mediated p62 cleavage at D256 under TNFα or LPS toxicity, and such cleavage appeared to play a key role to downgrade p62-droplet formation. Caspases are classified as initiator caspases and executioner caspases [62]. Although classified as an executioner, caspase-6 may be both upstream and downstream of the apoptotic cell death pathway. Caspase-6 is a direct activator for the initiator caspase-8 [63], and it cleaves the BH3-only protein Bid to sustain a feedforward caspase cascade in hepatocytes [64]. We proceeded to characterize the functional consequence of caspase-6-mediated p62 cleavage.

### Caspase-6-mediated p62 N-terminal cleavage fragment colocalizes with full-length p62 via the PB1 domain

**Fig. 5:**
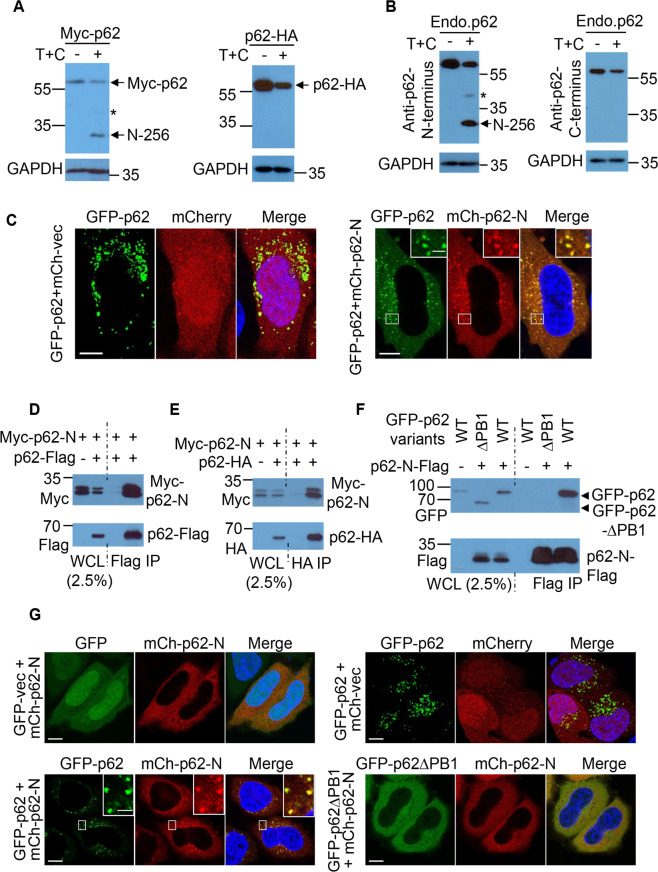
Caspase-6-mediated p62 N-terminal cleavage fragment colocalizes with full-length p62 via the PB1 domain. **A, B** Caspase-6-mediated p62 C-terminal fragment was undetectable. **A** N-terminally Myc-tagged p62 (Myc-p62) or C-terminally HA-tagged p62 (p62-HA) was transfected into HeLa cells. After 20 h, the cells was subjected to cleavage with the treatment of TNFα (10 ng/ml) + CHX (50 µg/ml) (T + C) for 4 h. The cell lysates were used for immunoblot with anti-Myc or HA antibody, and GAPDH antibody successively. An asterisk symbol (*) denotes Myc-p62-1-329aa cleavage fragment (left). N-256: Myc-p62 N-terminus 1-256aa (left). **B** HeLa cells was subjected to cleavage with the treatment of TNFα (10 ng/ml) + CHX (50 µg/ml) for 4 h. The cell lysates were used for immunoblot with anti-p62 N-terminus antibody or anti-p62-C-terminus antibody, and GAPDH antibody successively. An asterisk symbol (*) denotes p62-1-329aa cleavage fragment (left). N-256: p62 N-terminus 1-256aa (left). **C** The colocalization between mCherry-p62-N and GFP-p62. Hela cells expressing GFP-p62 and mCherry-p62-N were fixed, and images were acquired with Leica confocal microscopy. Bar: 10 µm. Boxed areas are magnified. Bar (inset): 2 µm. **D, E** The physical interaction between p62 and p62-N. **D** Myc-p62-N/vector or Myc-p62-N/p62-Flag were transfected into HeLa cells. After 20 h, cells were lysed and subjected to immunoprecipitation (IP) with anti-Flag (M2) agarose beads (Sigma). The immunoprecipitates and whole cell lysates (WCL) were used for immunoblot with anti-Myc and anti-Flag antibody successively. **E** Myc-p62-N/vector or Myc-p62-N/p62-HA were transfected into HeLa cells. After 20 h, cells were lysed and subjected to immunoprecipitation with anti-HA agarose beads (Sigma). The immunoprecipitates (IP) and whole cell lysates (WCL) were used for immunoblot with anti-Myc and anti-HA antibody successively. **F**, **G** The PB1 domain was required for the interaction between p62 and p62-N. **F** GFP-p62/vector, GFP-p62ΔPB1/p62-N-Flag or GFP-p62/p62-N-Flag were transfected into HeLa cells. After 20 h, cells were lysed and subjected to immunoprecipitation (IP) with anti-Flag (M2) agarose beads (Sigma). The immunoprecipitates and whole cell lysates (WCL) were used for immunoblot with anti-GFP and anti-Flag antibody successively. **G** Hela cells were transfected with GFP vector/mCherry-p62-N (control), GFP-p62/mCherry vector (control), GFP-p62/mCherry-p62-N or GFP-p62ΔPB1/mCherry-p62-N. After 20 h, cells were fixed and images were acquired with Leica confocal microscopy. Bar: 10 µm. No significant colocalization between GFP-p62ΔPB1 and mCherry-p62-N was observed. Boxed areas are magnified. Bar (inset): 2 µm.

We aimed to investigate the potential role of N-terminal (p62-N) and C-terminal p62 fragment (p62-C) in p62-droplet formation. We initially employed N-terminally Myc- or GFP-tagged p62, and C-terminally HA- or Flag-tagged p62 to test the protein levels of p62-N or p62-C. Interestingly, although p62-N was relatively stable, p62-C was undetectable under TNFα + CHX treatment (Fig. 5A and Fig. S5). Similarly, endogenous p62-C was also undetectable in the conditions (Fig. 5B). This suggests that p62-C was unstable in the cells with TNFα toxicity. We therefore focused on p62-N for subsequent functional characterization.

p62-N does not contain the UBA domain that is vital for p62-droplet formation [26, 36, 39], therefore p62-N is predictably unable to form droplets. However, full-length p62 and p62-N appeared to colocalize in p62 droplets (Fig. 5C). Indeed, p62-N physically interacted with full-length p62. Myc-p62-N was pulled down by immunoprecipitation (IP) of full-length p62-Flag with anti-Flag antibody (Fig. 5D) or p62-HA with anti-HA antibody (Fig. 5E). The PB1 domain is required for p62 oligomerization [27]. The PB1 domain may mediate the interaction between p62 and p62-N. We confirmed this by examining the interaction between p62-N and p62ΔPB1 (PB1-deleted p62, p62 121-440aa). Although wild-type p62 was pulled down by p62-N, the interaction between p62ΔPB1 and p62-N was not detected (Fig. 5F). Similarly, the colocalization between p62ΔPB1 and p62-N, in contrast with that between wild-type p62 and p62-N, was abolished (Fig. 5G). Loss of the interaction between p62ΔPB1 and p62-N validates that the PB1 domain is critical to mediate the p62-N-p62 interaction.

### Caspase-6-mediated N-terminal p62 cleavage fragment plays a dominant-negative role in p62-droplet formation

**Fig. 6:**
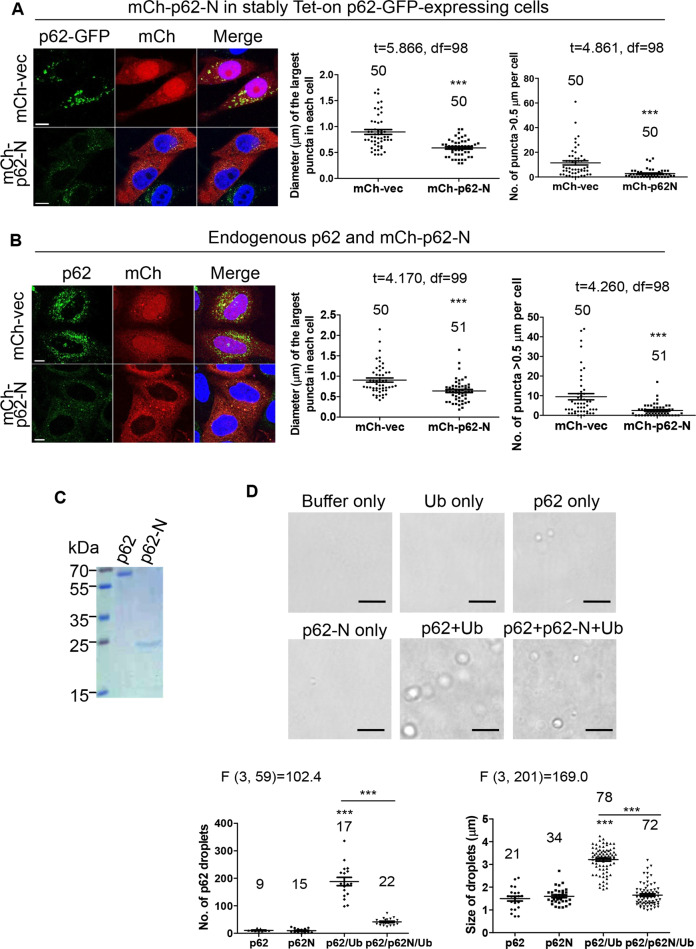
Caspase-6-mediated N-terminal p62 cleavage fragment plays a dominant-negative role in p62-droplet formation. **A, B** Caspase-6-mediated p62 cleavage blocked p62-droplet formation. **A** HeLa cells stably Tet-on expressing p62-GFP were transfected with vector or mCherry-p62-N (mCh-p62). p62-GFP-droplet formation was assessed (see below). **B** HeLa cells transfected with vector or mCh-p62-N, were stained with anti-p62 (C-terminus) antibody (guinea pig) for endogenous p62 proteins. Note that anti-p62 C-terminus antibody does not react with mCh-p62-N. Endogenous p62-droplet formation was assessed as below. Confocal images were acquired. Bar: 10 µm. The diameter of the biggest p62 puncta (µm) in each cell and the number of p62 puncta > 0.5 µm in each cell were assessed by ImageJ. *n* = the number of cells, as shown in each plot. Data are shown as mean ± sem. Statistical analysis was performed by unpaired/two-tailed T-test. ***P < 0.0001. **C** Coomassie blue staining of the purified bacteria-expressed His-tagged p62 and p62-N. **D** Caspase-6-mediated p62 cleavage fragment (p62-N) inhibited p62-droplet formation in vitro. Upper panels: The indicated individual proteins, or the multiple protein mixtures were subjected to in vitro phase separation in a microcentrifuge tube for 3 h in the buffer: 40 mM Tris-HCl, pH 7.4, 150 mM NaCl, 1 mM DTT, 10% glycerol. The phase separation buffer only was used as a control. Where appropriate, 3 µM final concentration of p62 was maintained; 3 µM final concentration of p62-N, or 1 µM final concentration of polyubiquitinated proteins (Ub) was added. For the phase separation of the mixture of p62, p62-N and isolated polyubiquitinated proteins, p62 and p62-N were mixed and incubated for 1 h prior to the addition of polyubiquitinated proteins (Ub). Imaging was acquired on a glass-bottomed 384-well plate. The phase-contrast images were acquired with Leica DMi8 microscopy. Scale bar: 10 µm. Lower panels: The number of p62 droplets in each image (232 µm × 310 µm) was scored (LAS-X). *n* = 9–22 images for each group, as indicated in the plot. The size of p62 droplets was assessed (LAS-X). *n* = 21–78 droplets, as indicated in the plot. Data are shown as mean ± sem. Statistical analysis was performed by one-way ANOVA with Bonferroni multiple comparison test. The F/degree of freedom/post hoc *P* values are indicated in each plot. ***P < 0.0001.

We tested if p62-N could modulate p62-droplet formation, and found that p62-N blocked full-length p62-droplet formation in GFP-p62-overexpressing cells (Fig. S6A), stably Tet-on p62-GFP-expressing cells (Fig. 6A) and the cells with endogenous full-length p62 (Fig. 6B). Moreover, p62-N reduced droplet formation of full-length p62 in both autophagy-competent, wild-type mouse embryonic fibroblasts (MEFs) and autophagy-defective, ATG5 KO MEFs (Fig. S6B), indicating that the effect of p62-N on full-length p62-droplet formation is independent of autophagy activity. Therefore, p62-N cleavage fragment appeared to block p62-droplet formation in a dominant-negative manner possibly through its physical interaction with full-length p62. These data suggest that inflammatory toxic stimuli, which activate caspase-6, produce dominant-negative p62-N to block p62-droplet formation. To further validate the dominant-negative role of p62-N in p62-droplet formation, we purified recombinant His-tagged full-length p62 and p62-N expressed in *E. coli* using 6× histidine affinity chromatography (Fig. 6C). As we have previously shown [37], recombinant p62 alone was insufficient for droplet formation (Fig. 6D). Polyubiquitinated protein binding to p62 UBA domain stimulates p62-droplet formation [36, 39]. Addition of purified polyubiquitinated proteins induced substantial p62-droplet formation (Fig. 6D). However, p62-N largely prevented polyubiquitinated proteins induced p62-droplet formation (Fig. 6D). These in vitro p62 phase separation assays validated that p62-N lacking UBA domain directly blocked p62-droplet formation, likely through its oligomerization with full-length p62, which attenuates the quantity of polyubiquitinated proteins able to bind full-length p62 in the p62-p62-N oligomeric unit (as compared to that in the p62-p62 oligomeric unit), leading to reduced p62-droplet formation.

### Caspase-6-mediated p62 cleavage negatively regulates the number and size of autophagosomes

p62 is well known to recruit protein cargo for its selective autophagic clearance, and importantly p62 droplets directly serve as platforms for autophagosome formation [40, 41]. p62 droplets were recently shown to support autophagosome formation through surface tension. Autophagosomes could initiate from phase-separated p62 droplets through droplet surface tension, and autophagosome completion may be led through droplet wetting and subsequent autophagosomal sheet bending [41]. The droplet size that influences the critical surface tension could also contribute to autophagosome formation. Given that caspase-6-mediated p62 cleavage fragment served a dominant-negative role in p62-droplet formation, it could modulate autophagy through autophagosome biogenesis, in addition to its regulation of p62 droplets based autophagic clearance of protein cargos. We hypothesized that the size of p62 droplets might influence autophagosome size and number.

**Fig. 7:**
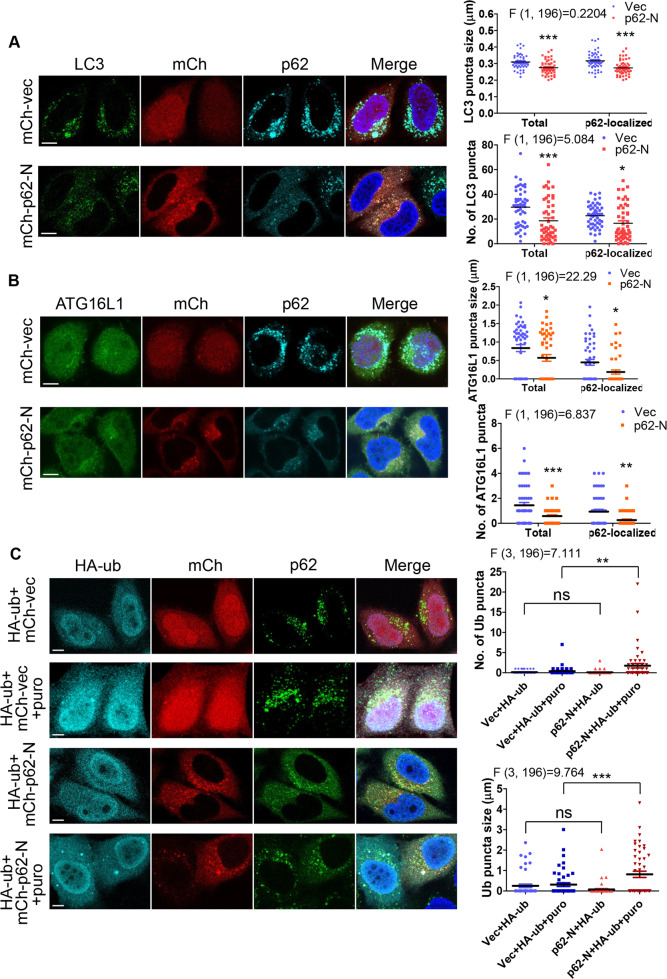
Caspase-6-mediated N-terminal p62 cleavage fragment negatively regulates p62 droplets associated autophagosome formation. **A** Caspase-6-mediated N-terminal p62 cleavage fragment reduced LC3-positive puncta formation. Cells transfected with mCherry empty vector (mCh-vec) or mCherry-p62-N (mCh-p62-N) were stained with anti-LC3 and p62 (C-terminus) antibody (guinea pig) for endogenous LC3 and p62 proteins. Note that anti-p62 C-terminus antibody does not react with mCh-p62-N. Confocal images were acquired. Bar: 10 µm. Total LC3 puncta in each cell or p62 droplets associated LC3 puncta in each cell were quantified. The diameter and the count of puncta in each cell were assessed by ImageJ. For the size of LC3 puncta, each point in the plot represents the average size of LC3 puncta in each cell. *n* = 50 cells from three independently plated wells. More than 10,000 LC3 speckles were analyzed. Data are shown as mean ± sem. Statistical analysis was performed by Two-way ANOVA with Bonferroni post-tests. The F/degree of freedom/post hoc *P* values are indicated in each plot. *P < 0.05; ***P < 0.0001. **B** Caspase-6-mediated N-terminal p62 cleavage fragment reduced ATG16L1-positive puncta formation. Cells transfected with mCherry empty vector (mCh-vec) or mCherry-p62-N (mCh-p62-N) were stained with anti-ATG16L1 and anti-p62 (C-terminus) antibody (guinea pig) for endogenous ATG16L1 and p62 proteins. Note that anti-p62 C-terminus antibody does not react with mCh-p62-N. Confocal images were acquired. Bar: 10 µm. Total ATG16L1 puncta in each cell or p62 droplets associated ATG16L1 puncta in each cell were quantified. The diameter and the count of puncta in each cell were assessed by ImageJ. For the size of ATG16L1 puncta, each point in the plot represents the average size of ATG16L1 puncta in each cell. *n* = 50 cells from three independently plated wells. Data are shown as mean ± sem. Statistical analysis was performed by Two-way ANOVA with Bonferroni post-tests. The F/degree of freedom/post hoc *P* values are indicated in each plot. *P < 0.05; **P < 0.01; ***P < 0.0001. **C** Caspase-6-mediated N-terminal p62 cleavage fragment enhanced the levels of puromycin-induced ubiquitin puncta. HeLa cells were transfected with mCherry empty vector /HA-ubiquitin or mCherry-p62-N/HA-ubiquitin (HA-ub). After 20 h, cells were treated with vehicle or puromycin (5 µg/ml) for 5 h. Cells were stained with anti-HA and p62 (C-terminus) (guinea pig) antibody. Note that anti-p62 C-terminus antibody does not react with mCh-p62-N. Confocal images were acquired. Bar: 10 µm. The diameter and the count of puncta in each cell were quantified by ImageJ. *n* = 50 cells from three independently plated wells. Data are shown as mean ± sem. Statistical analysis was performed by one-way ANOVA with Bonferroni multiple comparison test. The F/degree of freedom/post hoc *P* values are indicated in each plot. ns not significant; **P < 0.01; ***P < 0.0001.

We examined if caspase-6-mediated p62 cleavage reduced p62 droplets dependent autophagosome formation, and observed that the number and size of LC3 autophagic vesicles associated with p62 droplets were significantly decreased by p62-N (Fig. 7A), which lacks the LIR domain. Furthermore, the number and size of overall LC3-decorated autophagic vesicles were significantly reduced in the cells expressing p62-N (Fig. 7A). Consistently, the formation of ATG16L1 associated with p62 droplets was also weakened by p62-N, and the number and size of total ATG16L1 vesicles also declined in p62-N expressing cells (Fig. 7B). Unlike the fact that all the cells exhibited p62 droplets associated LC3 puncta, a proportion of cells did not have p62 droplets associated ATG16L1 vesicles. This allowed us to quantify the percentage of cells with ATG16L1 vesicles associated with p62 droplets, in the absence or the presence of p62-N. Indeed, the rate of the cells with p62 droplets associated ATG16L1 puncta was markedly lowered by p62-N (Fig. S7). We tested if the effect of caspase-6-mediated p62 N-terminal cleavage fragment is dependent on the presence of p62. To this end, we examined if p62-N modulated the levels of LC3 or ATG16L1 in WT or p62 KO MEFs. As shown in HeLa cells (Fig. 7A, B), both ATG16L1 (Fig. S8) and LC3 (Fig. S9A) vesicle formation was significantly reduced by p62-N in WT MEFs, but not in p62 KO MEFs. This suggests that the effect of p62-N on autophagic activity is dependent on the presence of p62. Interestingly, ATG16L1 vesicle formation was markedly reduced in p62 KO MEFs, compared to WT MEFs (Fig. S8); consistently in p62 KO MEFs, LC3 vesicle formation also dropped at a level lower than the detection threshold of our mouse LC3 monoclonal antibody (Fig. S9A). As reported previously [65], we confirmed that the LC3-II level in p62 KO MEFs were markedly reduced, compared to that in WT MEFs (Fig. S9B). These data are consistent with the notion that p62 droplets are critical for autophagosome formation [40, 41]. Interestingly, rapamycin treatment abolished the dominant-negative effect of p62-N on LC3 vesicle formation (Fig. S10), suggesting that inhibition of mTOR as a master regulator may stimulate autophagosome formation independently of p62 droplets.

We further investigated if caspase-6-mediated p62 cleavage exerted an effect on the clearance of polyubiquitinated proteins. Puromycin was used to induce formation of polyubiquitinated accumulates that are subsequently targeted for autophagic clearance. In basal conditions, p62-N appeared to marginally reduce the number and size of ubiquitin puncta (Fig. 7C, see column 1 and 3) that mark the levels of polyubiquitinated protein nucleation. p62-N markedly increased polyubiquitinated protein accumulation in the presence of puromycin (Fig. 7C), presumably due to the dominant-negative effect of p62-N on p62 droplets dependent autophagy that is required for the clearance of polyubiquitinated proteins. Mutant huntingtin (mHTT) is established to be a substrate of autophagy [66–68]. We confirmed that p62-N significantly enhanced mHTT aggregation in neuroblastoma SK-N-SH cells stably Tet-on expressing mHTT (Fig. S11). Our data indicate that inflammatory toxicity-elicited p62 cleavage by caspase-6 reduced the formation of p62 droplets that mediate selective autophagy as well as autophagosome formation (Fig. S12).

## Discussion

p62 signaling is regulated at both the transcriptional and the posttranslational level [42]. The regulation of p62-droplet formation is poorly understood. We initially observed that p62-droplet formation was selectively blocked by inflammatory toxicity. Mechanistically, p62 was cleaved at D256 by caspase-6 in inflammatory stress conditions. Caspase-6-mediated p62 C-terminal cleavage fragment appeared unstable, whereas the N-terminal fragment remained relatively stable, presumably due to its oligomerization with full-length p62 via the PB1 domain. As polyubiquitinated protein binding is required for p62-droplet formation [36, 39], p62-N lacking polyubiquitinated protein binding is predicted to reduce the quantity of polyubiquitinated proteins in the p62-p62-N complex, thereby exerting a dominant-negative effect on p62-droplet formation and subsequent p62 droplets based autophagy. Caspase-6 mediated p62 cleavage may also cause loss-of-function effects (due to reduction in full-length p62) on p62-droplet formation, in addition to its gain-of-function effects. The gain-of-function effects would be more important than loss-of-function ones, taking into account the fact that p62 levels are often elevated in stress conditions.

Autophagosomal sheet formation is supported by p62-droplet surface tension, and autophagosomal sequestration is accomplished by bending autophagosomal sheets to p62 droplets through the droplet-sheet adhesion strength. Consequently, the autophagosome selectively encloses p62-recruited cargos for autolysosomal clearance. With the surface tension of a droplet below or beyond its critical point, droplet autophagy can occur through the modes of piecemeal sequestration and complete sequestration, respectively. For the former scenario, piecemeal sequestration of a droplet occurs due to spontaneous sheet bending of the unstable sheets in the cup-shaped intermediate. In the latter, the sheets remain open until the critical size for sheet closure is reached for complete sequestration. The droplet size that influences the critical surface tension may also determine the modes of autophagosome formation. Droplets that are too small are unable to undergo piecemeal sequestration, while droplets that are too large are unable to undergo complete sequestration due to the limitations in membrane supply [41]. The appropriate size of a p62 droplet may be critical for autophagy regulation by determining autophagosomal biogenesis. Our study indicates that p62-N appeared to reduce the size and number of autophagosomes in cells, suggesting that p62-N reduces autophagosome synthesis potentially through a reduction in p62-droplet formation. In this study, we have not addressed if p62-N could affect autophagosome maturation, although such a possibility is less likely. Further investigation may elucidate if the caspase-6–p62 axis has a role in autophagosome maturation.

It was shown that proteolytic cleavage of p62 occurs at 329 by caspase-8 [57] and caspase-1 [56]. Upon the activation of Toll-like receptors, D329 cleavage by caspase-8 results in mTOR activation [57]. Caspase-1-induced p62 cleavage at D329 was reported to decrease IL-1β production, exerting an effect to balance caspase-1–induced inflammation [56]. Interestingly, the D329 cleavage site does not exist in mouse p62, while the D256 cleavage site of caspase-6 is conserved among zebrafish, mouse, rat and human p62. Compared to caspase-6 cleavage, D329 cleavage in p62 was a relatively minor event in human cells in our conditions (Figs. 2D, 3B–E, 4A, E and 5A, B, Fig. S5). Our study supports that caspase-6-mediated cleavage of p62 plays a critical role in negative regulation of p62 droplets based autophagy.

Although categorized as an executioner, the activation and function of caspase-6 remain poorly understood. Caspase-6 was reported to activate the initiator caspase-8 [63], and cleave the BH3-only protein Bid to mediate a feedforward loop to sustain the caspase cascade in hepatocytes [64]. Caspase-6 activation induces liver damage in nonalcoholic steatohepatitis [64]. Overall, 2% of 150 colonic or gastric cancers are associated with CASP6 mutations [69], and expression of caspase-6 in gastric cancer tissues is decreased [70]. These imply that caspase-6 would be critical for cellular homeostasis in the cells with high levels of inflammatory response. Dominant-negative regulation of p62 droplets based autophagy by inflammatory toxicity stress may have implications in a variety of pathological conditions. Many pathogens have evolved to block the prosurvival signal cascades of host cells for the benefit of pathogen survival. Yersinia species bacteria utilize the effector protein YopJ to block activation of TAK1 and IKK for inhibition of NF-kB [60, 61], leading to inflammation and caspase activation, which could result in p62 cleavage at D256, yielding the dominant-negative effect of p62-N on p62 droplets based autophagy. During viral infection, production of proinflammatory cytokines could exert dominant-negative effects on p62 droplets based autophagy to affect both p62 selective autophagy and bulk autophagy. In neurodegeneration conditions, neuro-proteotoxicity-induced inflammation could also cause caspase-6-mediated autophagy inhibition through dominant-negative regulation of p62-droplet formation. Caspase-6 has been shown to be implicated in neurodegenerative conditions including Huntington’s and Alzheimer’s diseases [71–73]; p62 droplets based autophagy is key to clearance of toxic aggregation-prone proteins [8, 30, 46]. Caspase-6 cleavage of p62 is likely to be an important event to negatively regulate p62-mediated droplet autophagy in protein misfolding toxicity. Tackling the caspase-6–p62 axis could have therapeutic values for these conditions.

p62 also plays roles in cell survival, amino acid sensing and the oxidative stress response [74–76]. Caspase-6-mediated p62 cleavage could have functions other than its role in p62 droplets based autophagy. For instance, p62 interactions with RIP, aPKC and TRAF6 activate TRAF6 and subsequently result in the activation of NF-κB during intereukin-1, RANKL or NGF stimulation [74]. In oxidative stress conditions, p62 recruits Keap1 from the cytoplasmic Keap1-Nrf2 complex. As a result, freed Nrf2 translocates into the nucleus as a transcription factor, inducing a battery of Nrf2 target genes encoding antioxidant and anti-inflammatory enzymes [76, 77]. It is unknown whether caspase-6-mediated p62 cleavage is involved in these pathways. On the other hand, proteotoxicity may result in upregulation of p62 expression [6, 45, 78–80], potentially leading to unconstrained p62 aggresome formation. The dominant-negative role of caspase-6-mediated p62 cleavage could limit p62 aggresome formation and toxicity in proteotoxic conditions. Further studies may provide insight into the additional roles of the caspase-6–p62 axis in these conditions.

## Materials and methods

### Antibodies and reagents

The indicated antibody dilutions were used for western blot (otherwise indicated). Rabbit polyclonal antibodies: anti-caspase-3 (1:1000) (CST, #9662); anti-cleaved caspase-3 (1:1000) (CST, #9661); anti-caspase-6, (1:1,000) (CST, #9762); anti-caspase-8 (1:1000) (CST, #9746); anti-caspase-9 (1:1000) (CST, #9504); LC3A/B (1:1000) (CST, #12741); anti-p62 (1:3000) (MBL, #PM045); anti-p62 (1-250 aa) (1:500) (Abcam, #ab240635); anti-Flag (1:1000) (CST, #14793); anti-GFP (1:1000) (CST, #2956); anti-β-actin (1:1000) (Thermo, #RB-9421); anti-Myc (1:1000) (CST, #2272); anti-ATG16L1 (MBL, #PM040). Anti-mouse monoclonal antibodies: anti-GAPDH (1:5000) (Ambion, #AM4300); anti-LC3 (MBL, #M152); anti-HA (1:1000) (BioLegend, #901501). Anti-HA agarose affinity gel (#A2095) and anti-Flag (M2) agarose affinity gel (#A2220) were from Sigma. Guinea pig polyclonal antibodies: anti-p62 (C-terminus) (1:2000) (Progen, #GP62-C).

Human TNFα was from Invitrogen (Sino Biological, #10602-HNAE-5). Mouse TNFα was from Peprotech. Recombinant human caspase-6 was from Novus (#NBP1-99589). Recombinant human caspase-3 (#CC119) and human caspase-9 (#CC120) were from Chemicon. Recombinant p62 (86-440aa) protein was purchased from Abcam (#ab95320). z-VAD-fmk (z-VAD) (#627610), caspase-3 inhibitor (Ac-DEVD-CHO) (#235420), calpastatin (#208902), granzyme B inhibitor (#366055) and PI-103 (#528100) were from Calbiochem (now Merck); caspase-6 inhibitor (Z-VEID-fmk) (#fmk006) was purchased from Biotechne. Doxycycline hydrochloride (#10592-12-9) was from Fisher Bioreagents; cycloheximide (#C4859), staurosporine (#S5921), chloroquine (C6628) and hygromycin B (#H3274) were from Sigma. G418 (#11811023) was a product of Thermo Fisher. Puromycin (#53-79-2) was obtained from Santa Cruz. IKK-16 (#ab216471) was from Abcam. Rapamycin (#R0395), MG132 (#M8699) and etoposide (#E1383) were from Sigma. Doxycycline hydrochloride (#10592-12-9) was from Fisher Bioreagents. siRNAs were from Invitrogen (Ambion) or Eurofins. Recombinant caspase-6 were purchased from Biotechne.

### DNA constructs

**Table:** 

Plasmids used in the study	Vectors (cut sites 5′/3′)	Inserts (cut sites 5′/3′)
pEGFP-C1-p62	pEGFP-C1 (BamHI/EcoRI)	p62 (BglII/EcoRI)
pEGFP-C1-p62-1-329aa	pEGFP-C1 (BamHI/EcoRI)	p62 1-329(BglII/EcoRI)
pEGFP-C1-p62-1-370aa	pEGFP-C1 (BamHI/EcoRI)	p62 1-370 (BglII/EcoRI)
pEGFP-C1-p62-121-440 (p62-ΔPB1)	pEGFP-C1 (BamHI/EcoRI)	p62-121-440 (p62-ΔPB1) (BglII/EcoRI)
pCMV-6M (Myc)-p62	pCMV-6M (BamHI/EcoRI)	p62 (BglII/EcoRI)
pCMV-6M (Myc)-p62-1-329aa	pCMV-6M (BamHI/EcoRI)	p62 1-329(BglII/EcoRI)
pCMV-6M (Myc)-p62-1-370aa	pCMV-6M (BamHI/EcoRI)	p62 1-370 (BglII/EcoRI)
pmCherry-p62	pcDNA3 mCherry (BamHI/EcoRI)	p62 (BglII/EcoRI)
pcCMV5c-p62-Flag	pCMV-5c (EcoRI/NotI)	p62 (EcoRI/NotI)
pET28a-p62	pET28a (BamHI/NotI)	p62 (BglII/NotI)
pET28a-p62 1-256aa	pET28a (BamHI/EcoRI)	(BamHI/EcoRI)

The correct DNA sequences were confirmed by DNA sequencing. p62-HA (#28027) and pcDNA3 HA-ubiquitin (#18712) were from Addgene.

### Mutagenesis

Plasmids for p62 point mutants, pEGFP-C1-p62D256A, pEGFP-C1-p62D258A, pEGFP-C1-p62D256A D258A, pEGFP-C1-p62-N (1-256aa) were generated using the QuikChange Multi Site-directed Mutagenesis kit according to manufacturing instruction (Agilent Technologies, #200514). Mutations were confirmed by DNA sequencing.

### Primer sequences

#### Mutagenesis

p62D256A: 5′-TCTGGGCATTGAAGTTGCTATCGATGTGGAGCACG-3′.

p62D258A: 5′-CATTGAAGTTGATATCGCTGTGGAGCACGGAGGGA-3′.

p62D256A D258A: 5′-TGGGCATTGAAGTTGCTATCGCTGTGGAGCACGGAGG-3′.

p62-N256: 5′-CCTCTAGGCATTGAGGTTGACTGAGATGTGGAACATGGAGGGAAG-3′.

### PCR primers

Full-length p62: Forward: 5′-AGAGATCTATGGCGTCGCTCACCGTG-3′; Reverse: 5′-GA GAATTCTCACAACGGCGGGGGATGCTT-3′.

p62 121-440aa (p62-ΔPB1): Forward: 5′-TGAGATCTATGGTGCACCCCAATGTG-3′; Reverse: 5′-GA GAATTCTCACAACGGCGGGGGATGCTT-3′.

p62 1-370aa: Forward: 5′-AGAGATCTATGGCGTCGCTCACCGTG-3′; Reverse: 5′-TAGAATTCTTAGGGGGGGTCCAGAGAGCT-3’.

p62 1-329aa: Forward: 5′-AGAGATCTATGGCGTCGCTCACCGTG-3′; Reverse: 5′-CGGAATTCTTA ATCCGACTCCATCTGTTC-3′.

### siRNAs

siRNAs were purchased from the suppliers as indicated. Nontargeting siRNA was the control siRNA. Human siRNA sequences: control siRNA-1 (Eurofins): 5′-CGUACGCGGAAUACUUCGA-3′; caspase-3 siRNA (Sense: 5′-UGGAUUAUCCUGAGAUGGG-3′) (Invitrogen); caspase-6 siRNA (sense 5′-GGAUAUUAUUCUCACCGGG-3′); caspase-9: (Sense: 5′-GGUUCUCAGACCGGAAACA-3′) (Invitrogen).

### Cell culture

Hela (ATCC, #CCL-2) were cultured with standard methods in Dulbecco’s Modified Eagle’s Medium (DMEM) (D6046) supplemented with 10% (v/v) fetal bovine serum (FBS) (Gibco, 10500-064) and 1/100 (v/v) 100× penicillin-streptomycin-L-glutamine (Thermo Fisher, #10378016) (full DMEM media). p62 KO MEFs and control MEFs (kindly provided by Dr M. Komatsu) [40], ATG5 KO MEFs and control MEFs (kindly provided by Dr N. Mizushima) [81], were cultured in full DMEM media. BV2 mouse microglial cells (kindly provided by Dr H. Li, UCL) were cultured in DMEM-F12 supplemented with 10% FBS and 1/100 (v/v) 100× penicillin-streptomycin-L-glutamine. COLO-205 cells (CLS, #330380) were cultured in RPMI1640 supplemented with 10% FBS and 1/100 (v/v) 100× penicillin-streptomycin-L-glutamine. SK-N-SH stably Tet-on expressing GFP-HTT exon 1-72Q cells (generated in this study) were cultured in DMEM supplemented with 10% FBS containing 100 µg/ml G148 and 50 µg/ml Hygromycin B. For expression of GFP-HTT exon 1-72Q, the cells were induced with doxycycline (DOX) (250 ng/ml) for the time indicated. Stably Tet-on p62-GFP-expressing HeLa cells (generated in our laboratory) [37] were cultured in DMEM supplemented with 10% FBS containing 100 µg/ml G148 and 50 µg/ml Hygromycin B. p62-GFP expression was induced with 250 ng/ml DOX. Authentication was confirmed by the suppliers, and by morphology check with light microscopy. All the cell lines used were negative for mycoplasma.

### DNA and siRNA transfection

Cells were split 1 day prior to transfection to 50–80% confluency and left overnight in DMEM containing 10% FBS. DNA constructs and siRNAs were transfected with lipofectamine 2000 according to the manufacturer’s instructions. For a well of the six-well plate, 100 µl Opti-MEM containing transfected plasmids was mixed with 100 µl Opti-MEM with lipofectamine. After 15-min incubation, the transfection mixture was added to the cells, where the medium was pre-changed with 0.8 ml of antibiotics-free DMEM containing 10% FBS.

For DNA transfection, 0.1–0.3 µg of each plasmid was used for a well of a six-well plate, or the proportional amount of plasmids was transfected for a well of a non-six-well plate. 2 µl of lipofectamine 2000 reagent was used for each µg plasmid DNA. Media containing transfection reagent was changed with full DMEM media 4 h after transfection. Transfected cells were typically harvested or fixed 20 h post transfection.

For siRNA transfection, siRNAs were transfected at a final concentration of 50 nM, and 1 µl of lipofectamine 2000 reagent was used for each 20 picomole siRNA. HeLa cells were maintained in 10% FBS DMEM containing no antibiotics for 24 h after transfection. After 24 h, the siRNAs transfected cells were either split for subsequent experiments or were cultured continuously with full DMEM media until harvested or fixed for further analysis.

### Immunocytochemistry

Cells were fixed with 4% paraformaldehyde for 10 min. The fixed cells were washed three times in PBS, then permeabilized with PBS containing 0.5% Triton for 10 min. Cells were blocked in blocking buffer (1% BSA, 1% heat inactivated goat serum in PBS) for 30 min at room temperature. Primary antibodies were incubated with cells overnight at 4 °C. The secondary antibody was incubated for 30 min after washing three times with PBS (10 min, each). Cells were washed three times (10 min, each) after incubation with secondary antibodies, then mounted with DAPI (1 µg/ml). Images were acquired using a Leica confocal microscope.

### Western blot analysis

Cells were lysed in buffer A (20 mM Tris-HCl, pH 7.2, 2 mM MgCl_2_, 150 mM NaCl, 0.5% NP-40) with the protease inhibitor (Thermo Scientific/Pierce, #A32953). Protein concentrations were measured with a BCA protein assay kit (Thermo Scientific/Pierce, #23225). Cell lysates or protein solutions were mixed with an equal volume of 2× Laemmli buffer, and boiled at 100 °C for 10 min. The boiled protein samples were subjected to 10% or 12% SDS-PAGE resolution, and subsequently transferred to the PVDF membrane (Thermo Scientific, #88518). The PVDF membrane was blocked in 5% (w/v) semi-skimmed milk in 1× TBS with 0.05% Tween-20 (TBST), and incubated with a primary antibody at 4 °C typically for overnight in TBST containing 5% milk, followed by 5-min washing, three times. The membrane was incubated with a secondary antibody (cross-linked with HRP) at room temperature for 30 min. After three-time washing (5 min each), protein bands on the membrane were detected with the ECL western blotting substrate (Thermo Scientific/Pierce, #32106 or GE, #RPN2232).

### Cell viability assay

Cell survival was determined with the CellTiter-Glo Luminescent Cell Viability Assay kit (Promega) to measure ATP levels according to the manufacture instruction. Briefly, cells in a 96-well plate were treated with agents in the conditions indicated in each experiment. One hundred microliters of the Cell Titer-Glo reagent was added to a well with cells in 100 µl culture medium. Cells were placed on a shaker for 2 min and then incubated at room temperature for 10 min. The plate was read with a microplate reader (BMG Labtech) for luminescence measurement.

### Particle analysis for protein puncta

For microscopy data collection, the objects were randomly chosen. The sizes and numbers of p62, LC3, ATG16L1 or ubiquitin puncta were measured with ImageJ (particle analysis). Single-channel images were exported, and the scale was set by drawing a line paralleling to the scale bar. Images were processed with the despeckle function to decrease the noise, and a threshold was set to highlight puncta. Cells were selected by the freehand drawing tool. The analyze-particle function was initiated for the sizes and numbers of the vesicles. For the analysis of LC3 or ATG16L1 vesicles associated with p62 droplets, the vesicles that were not associated with p62 droplets were deleted, and the numbers and sizes of p62 droplets associated vesicles were achieved by the particle-analysis function of ImageJ. The size of LC3, ATG16L1 or ubiquitin puncta in each cell was pre-averaged. Typically 50 cells (n number) were analyzed. The analysis was conducted in a blinded manner by a researcher who was unaware of sample labeling and expected outcomes.

### 6× His-tagged protein expression and purification

p62 or p62-N was cloned into pET28a (Novagen) for 6× His-tagged protein expression. A plasmid was transformed into BL21 (DE3). A final concentration of 0.2 mM IPTG was added to LB broth to induce recombinant protein expression. 6× His-tagged proteins were purified with Ni^2+^-charged 6× His-tag affinity resins (Millipore) according to manufacture instruction. Briefly, the pellets from 250 ml bacterial culture were resuspended in 12 ml of binding buffer (50 mM NaH_2_PO_4_, pH 8.0, 300 mM NaCl, 10 mM imidazole) containing protease inhibitor cocktail. The cells were lysed by adding 1.2 ml of Bugbuster 10× protein extraction reagent (Millipore), 12 µl benzonase nuclease (250 U/µl) (Thermo, #88701) and 12 µl (50 mg/ml) lysozyme (Thermo, #90082). After centrifugation at 16,000 × *g*, 20 min, the supernatants were applied to 2 ml of 6× His-affinity beads. The beads were washed with 20 ml wash buffer (50 mM NaH_2_PO_4_, pH 8.0, 300 mM NaCl, 20 mM imidazole), three times, and eluted with 3 ml elution buffer (50 mM NaH_2_PO_4_, pH 8.0, 300 mM NaCl, 250 mM imidazole). The elution buffer was exchanged with the desired stock buffer (40 mM Tris, pH 7.4, 300 mM NaCl, 1 mM DTT, 10% glycerol, or 40 mM Tris, pH 7.4, 1 mM DTT, 10% glycerol) using a 4-ml EMD Amicon Ultra centrifugal filter unit (Millipore). Protein concentration was measured by BCA assays.

### Immunoprecipitation

IP was performed using buffer A (20 mM Tris-HCl, pH 7.4, 2 mM MgCl_2_, 150 mM NaCl, 0.5% NP-40, protease inhibitor cocktail (Roche)). Cells were lysed in 300–350 µl buffer A for 15 min on ice, followed by centrifugation at 13,000 × *g* for 15 min. 500 µg–1 mg total protein were used as the starting material for IPs. Ten microliters of pre-buffer A-washed anti-Flag antibody (M2) agarose affinity gel (Sigma) or anti-HA antibody agarose affinity gel (Sigma) was added the protein lysate and incubated for 2 h at 4 °C. IP products were directly boiled in Laemmli buffer or stored in −80 °C for western blot analysis.

### Polyubiquitinated protein purification

HA-ubiquitin plasmid (pcDNA-HA-ubiquitin) was transfected into HeLa cells in 3 × 10-cm dish. After 20 h, the cells were treated with puromycin (5 µg/µl, 5 h) to enrich HA-polyubiquitinated proteins. HA-polyubiquitinated proteins were subjected to IP with 40 µl anti-HA agarose beads (Sigma). The pull-down products were eluted with 120 µl of 0.1 M glycine (pH 2.5) by 10-min incubation at room temperature. The eluate was neutralized with 30 µl of 1 M Tris-HCl pH 7.4. The purified polyubiquitinated proteins were measured for concentration by BCA assays, and aliquoted for in vitro p62 phase separation assays. Protein molar concentration was estimated using the averaged molecular weight of 40 kDa.

### In vitro phase separation

For in vitro phase separation, purified recombinant p62 or p62-N was centrifuged at 16,000 × *g* for 5 min to remove any potential protein aggregates. p62-droplet phase separation was carried out in a glass-bottomed well of a 384-well plate in the buffer containing 40 mM Tris-HCl, pH 7.4, 150 mM NaCl, 1 mM DTT and 10% glycerol at room temperature. p62 was stocked in the buffer containing 40 mM Tris-HCl pH 7.4, 300 mM NaCl, 1 mM DTT and 10% glycerol. Purified recombinant p62-N was stocked in the buffer containing 40 mM Tris-HCl pH 7.4, 1 mM DTT and 10% glycerol. For in vitro phase separation, protein mixtures were adjusted to the phase separation buffer conditions, and the concentration of p62 or p62-N was kept consistent across the relevant samples. Images were acquired on a 384-well plate with Leica DMi8 microscopy.

### In vitro caspase cleavage

One microgram of recombinant p62 (86-440aa) (Abcam) was mixed with 1 U caspase-3 (Chemicon), 1 U caspase-6 (Novus) or 1 U caspase-9 (Chemicon) in the cleavage reaction buffer (50 mM HEPES pH 7.2, 50 mM NaCl, 0.1% CHAPs, 10 mM EDTA, 5% glycerol and 10 mM DTT) for 1 h at 37 °C. The cleaved products were subjected to Coomassie blue staining and western blot analysis.

### p62 protein sequence alignment

p62 protein sequences from different species were aligned with the T-COFFEE server (http://tcoffee.crg.cat/apps/tcoffee/do:mcoffee).

### Quantification of protein levels from western blot analysis

To measure protein levels from western blot analysis, the intensities of protein bands were analyzed using ImageJ software. The relative value of a specific protein band intensity versus that of its loading control band was computed as the relative level of the specific protein in a sample.

### Statistical analysis

Sample sizes were determined based on our preliminary results or our/others’ published results in similar experiments. No data were excluded from the study. The variance was similar between the groups that were being statistically compared. Statistical analysis was performed with GraphPad Prism v5. The unpaired two-tailed *t*-tests were conducted for the comparison between two groups (***P < 0.001; **P < 0.01; *P < 0.05; ns not significant); one-way or two-way ANOVA tests were used for the comparison among multiple groups: one-way ANOVA for variables influenced by a single factor; two-way ANOVA for variables influence by two or more factors (***P < 0.0001; **P < 0.01; *P < 0.05; ns: not significant). Data from at least three independent experiments were analyzed.

### Reporting summary

Further information on research design is available in the Nature Research Reporting Summary linked to this article.

## Supplementary information

Supplementary information

Reporting summary

## Data Availability

The data that support the findings of this study are available from the corresponding author upon reasonable request.
