# Supplementary material for: The caspase-6–p62 axis modulates p62 droplets based autophagy in a dominant-negative manner
Source: Cell Death Differ. 2021 Dec 3;29(6):1211–27. doi: 10.1038/s41418-021-00912-x (PMC9178044; doi:10.1038/s41418-021-00912-x)
Supplement: Supplementary file 1 — Supplementary information [file 41418_2021_912_MOESM1_ESM.pdf]

Supplementary Information for

The caspase-6-p62 axis modulates p62 droplets-based autophagy in a dominant-negative manner

- Supplementary Figures 1-12

Figure S1

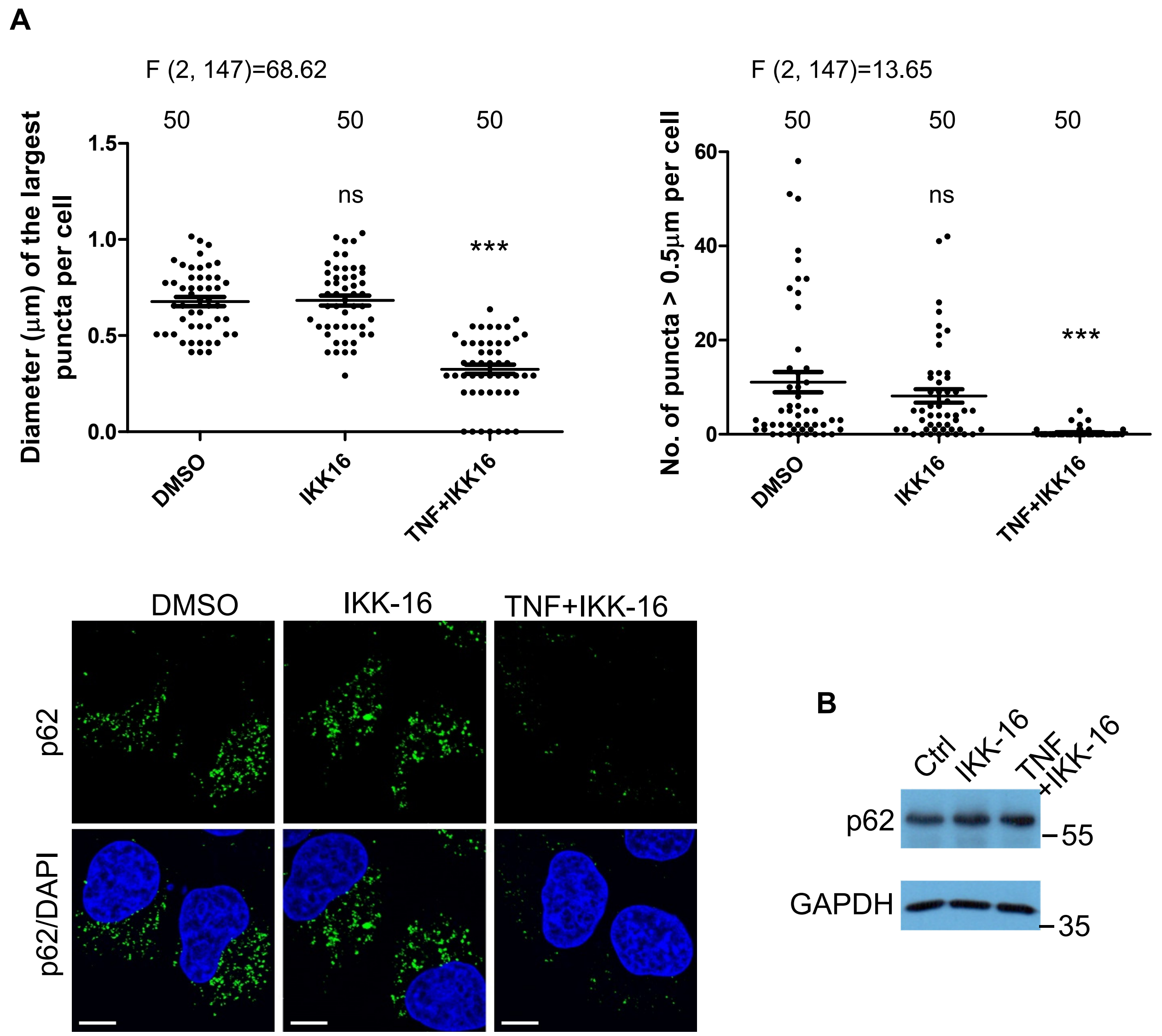

Figure S1. p62-droplet formation in TNFα-treated cells under NF-κB inhibition. HeLa cells were treated with vehicle (DMSO), IKK-16 (1 μM) (a selective inhibitor of IκB kinase (IKK) to block NF-κB activation), or TNFα (10 ng/ml) + IKK-16 (1 μM) for 3 hours. **(A)** The cells were stained with anti-p62 (guinea pig). Confocal images were acquired with confocal microscopy. Bar: 10 μm. The diameter of the biggest p62 puncta (μm) in each cell was measured (ImageJ), and the number of p62 puncta>0.5 μm in each cell was assessed (ImageJ). n= the number of cells, as shown in each plot. Data are shown as mean±sem. Statistical analysis was performed by One-way ANOVA with Bonferroni's Multiple Comparison Test. The F/degree of freedom/post-hoc p values is indicated in each plot. ns: not significant; ***: P<0.0001. **(B)** Full-length p62 levels in the cells under NF-κB inhibition. The cells treated as indicated were lysed and subjected to immunoblot with anti-p62 and GAPDH antibodies, respectively.

Figure S2

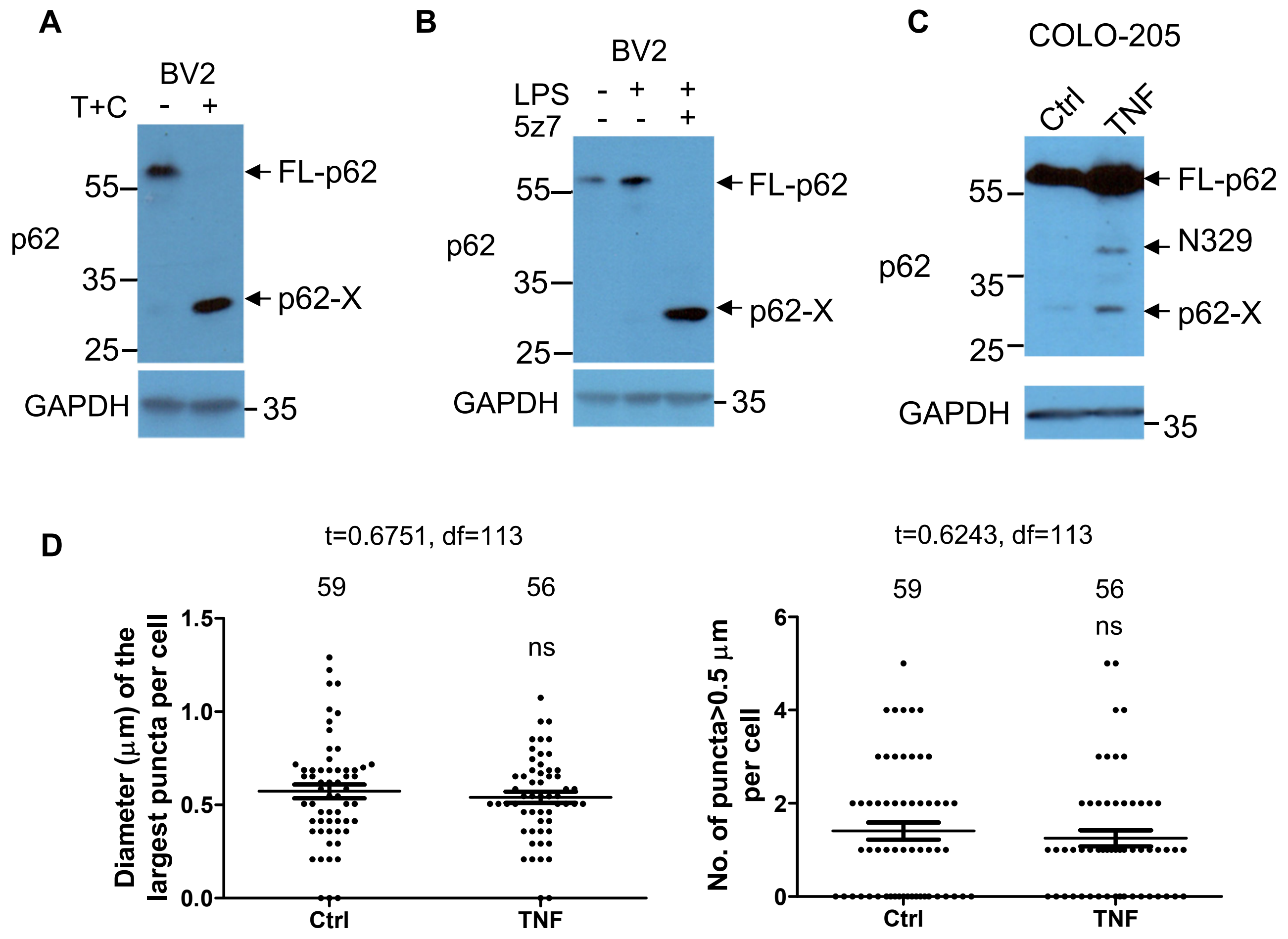

Figure S2. The new p62 species was produced in human or mouse cells under TNFα or LPS treatment. **(A)** Mouse BV2 cells were treated with TNFα (10 ng/ml)+CHX (50 μg/ml) (T+C) for 0 or 4 hours. The cell lysates were subjected to immunoblot with anti-p62 (N-terminus) and GAPDH antibodies successively. **(B)** Mouse BV2 cells were treated with the vehicle (DMSO), LPS or LPS+5z7 for 4 hours, and the cell lysates were subjected to anti-p62 (N-terminus) and GAPDH antibodies successively. **(C)** Human colon carcinoma COLO-205 cells were treated with the control (H2O) (Ctrl) or TNFα (30 ng/ml) for 6 hours. The cells were lysed and subjected to immunoblot with anti-p62 (N-terminus) and GAPDH successively. **(D)** The cells, treated with Ctrl (H2O) or TNFα (30 ng/ml) for 6 hours, were stained with anti-p62 (guinea pig). Confocal images were acquired with confocal microscopy. The diameter of the biggest p62 puncta (μm) in each cell was measured (ImageJ), and the number of p62 puncta>0.5 μm in each cell was assessed (ImageJ). n= the number of cells, as shown in each plot. Data are shown as mean±sem. Statistical analysis was performed by unpaired/two-tailed T-test. ns: not significant.

Figure S3

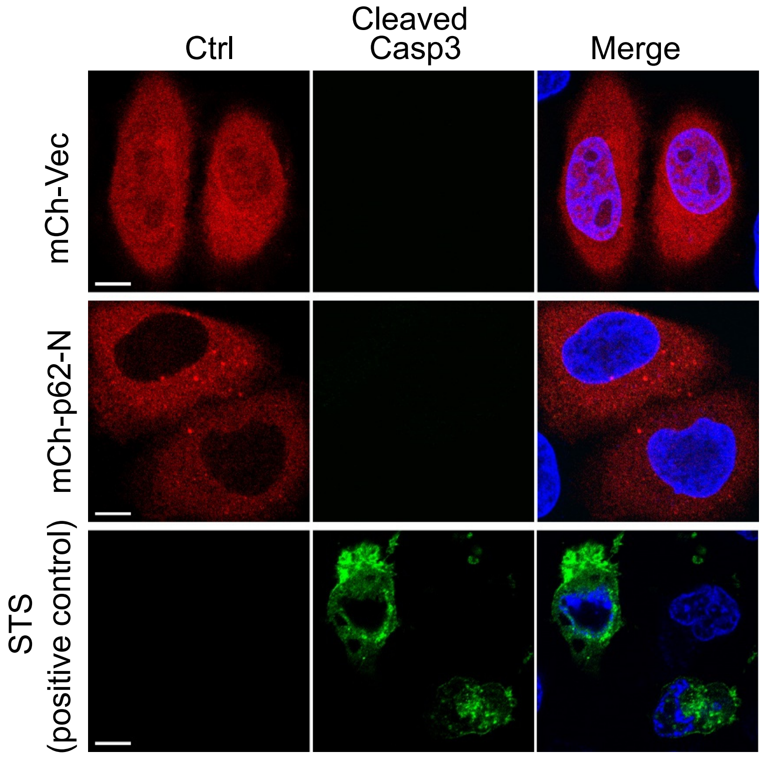

Figure S3. p62-N expression did not cause cytotoxicity. HeLa cells harbouring empty vector or p62-N were stained with anti-cleaved caspase-3 antibody. HeLa cells treated with staurosporine (STS) (1 μM) for 5 hours were also stained with the antibody for a positive control purpose. Images were acquired with confocal microscopy. Bar: 10 μm.

Figure S4

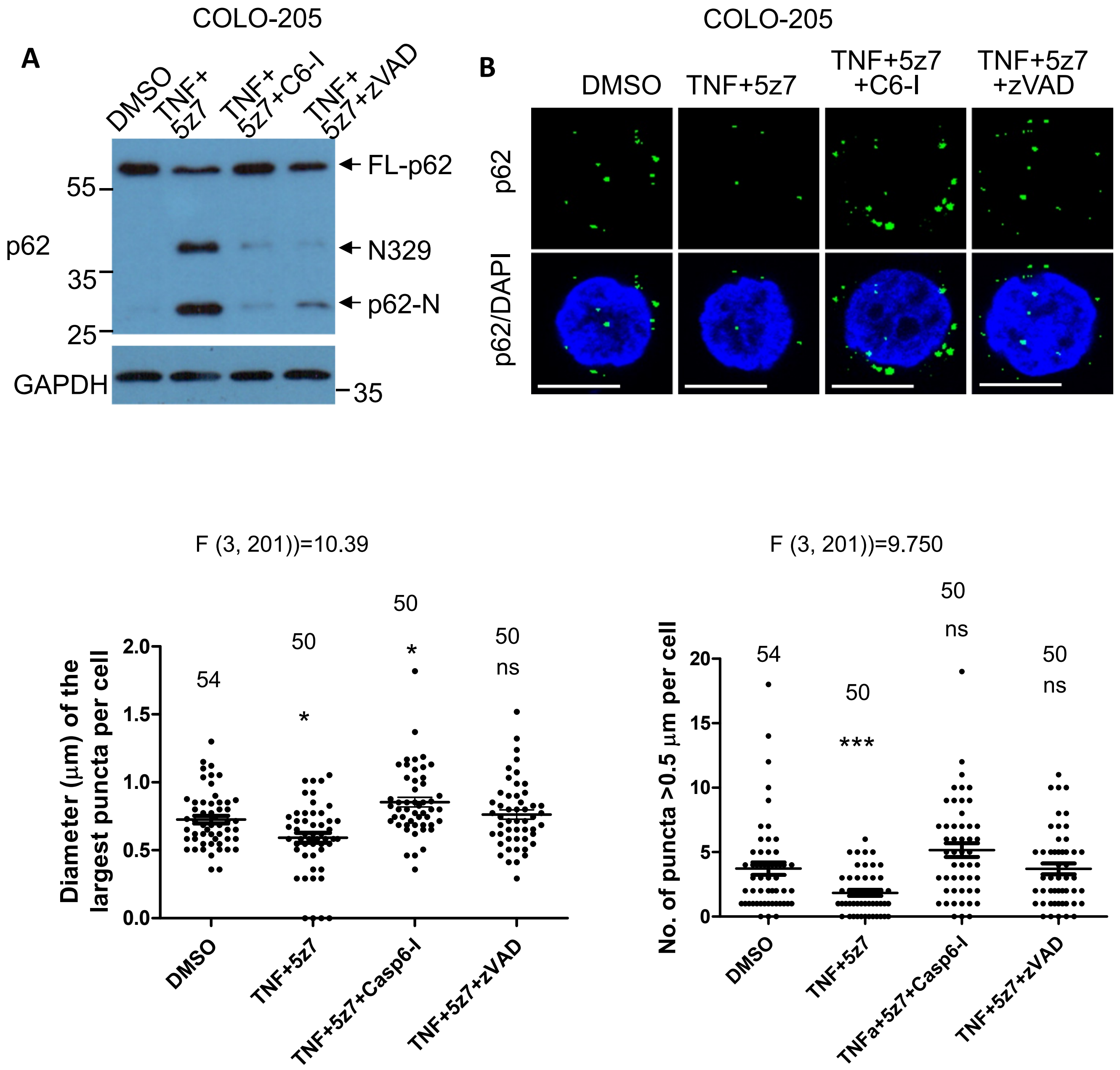

Figure S4. Caspase-6 inhibitor blocked p62 cleavage at D256 in COLO-205 cells. COLO-205 cells were treated with the vehicle (DMSO), TNFα (30 ng/ml) + 5Z-7-oxozeaenol (5z7) (0.5 μM), TNFα (30 ng/ml) + 5z7 (0.5 μM) + caspase-6 inhibitor (20 μM), or TNFα (30 ng/ml) + 5z7 (0.5 μM) + z-VAD-fmk (20 μM) for 4 hours. **(A)** The cell lysates were subjected to anti-p62 (N-terminus) and GAPDH successively. **(B)** The cells were stained with anti-p62 (guinea pig). Confocal images were acquired with confocal microscopy. Bar: 10 μm. The diameter of the biggest p62 puncta (μm) in each cell was measured (ImageJ), and the number of p62 puncta>0.5 μm in each cell was assessed (ImageJ). n= the number of cells, as shown in each plot. Data are shown as mean±sem. Statistical analysis was performed by One-way ANOVA with Bonferroni's Multiple Comparison Test. The F/degree of freedom/post-hoc p values is indicated in each plot. ns: not significant; *: $P<0.05$; ***: $P<0.0001$.

Figure S5

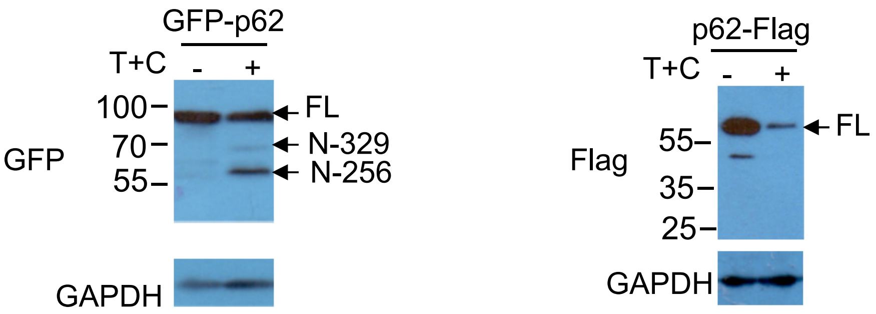

Figure S5. Caspase-6-mediated p62 C-terminal fragment was undetectable. N-terminally GFP tagged p62 (GFP-p62) or C-terminally Flag-tagged p62 (p62-Flag) was transfected into HeLa cells. After 20 hours, the cells was subjected to cleavage with the treatment of TNFα (10 ng/ml) + CHX (50 μg/ml) (T+C) for 4 hours. The cell lysates were used for immunoblot with anti-GFP or Flag antibody, and GAPDH antibody successively. N-329: GFP-p62 1-329; N-256: GFP-p62 1-256.

Figure S6

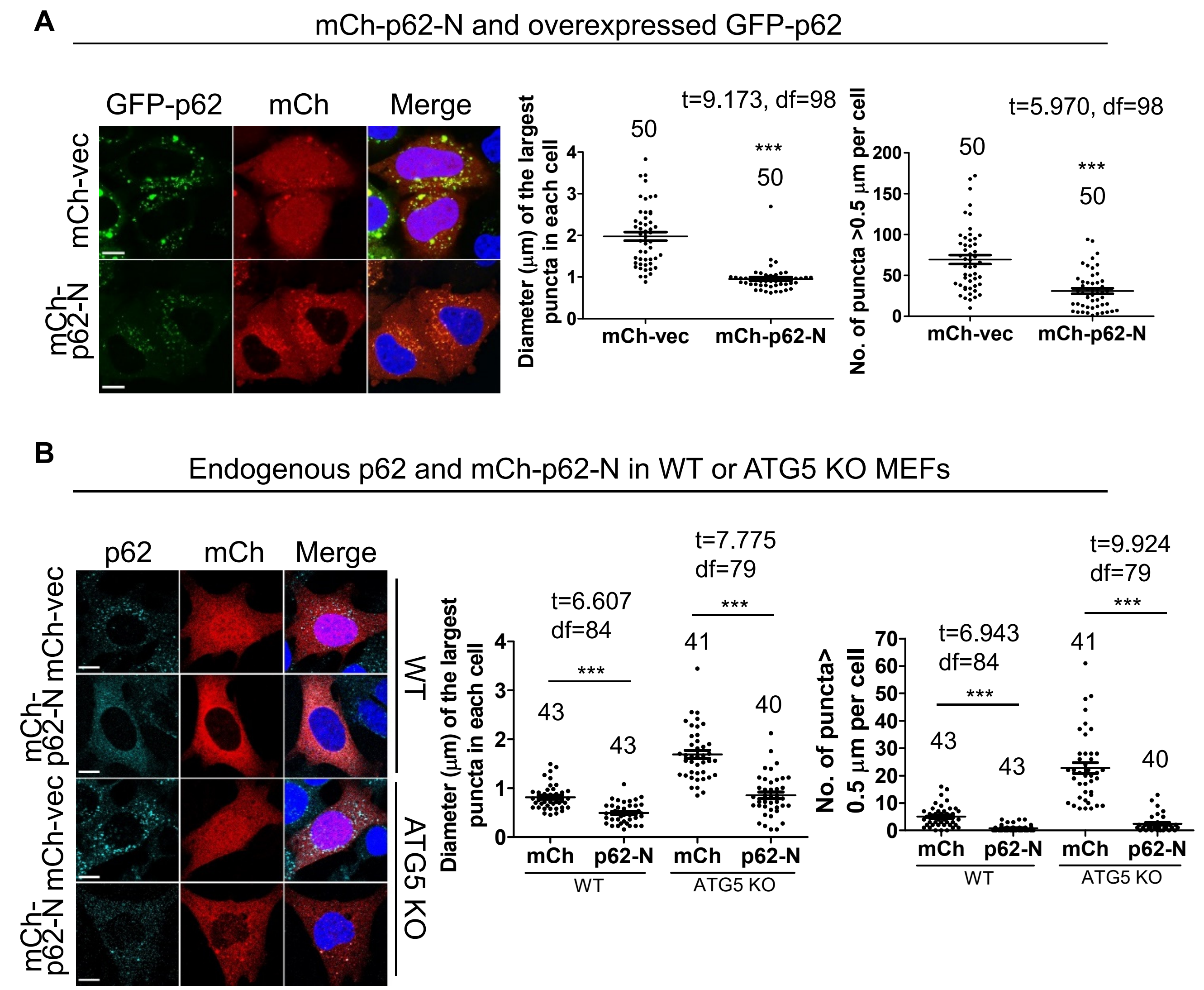

Figure S6. Caspase-6-mediated N-terminal p62 cleavage fragment played a dominant-negative role in p62 droplet formation. (A) HeLa cells were transfected with GFP-p62+mCherry vector or GFP-p62+mCherry-p62-N. GFP-p62 droplet formation was assessed (see below). (**B**) WT or ATG5 KO MEFs transfected with vector or mCherry-p62-N, were stained with anti-p62 (C-terminus) antibody for endogenous p62 signals. Note that anti-p62 C-terminus antibody does not react with mCh-p62-N. Endogenous p62 droplet formation was assessed as below. Confocal images were acquired. Bar: 10 μm. The diameter of the biggest p62 puncta (μm) in each cell and the number of p62 puncta>0.5 μm in each cell were assessed by ImageJ. n= the number of cells from 3 independently plated wells, as shown in each plot. Data are shown as mean±sem. Statistical analysis was performed by unpaired/two-tailed T-test. ***: P<0.0001.

Figure S7

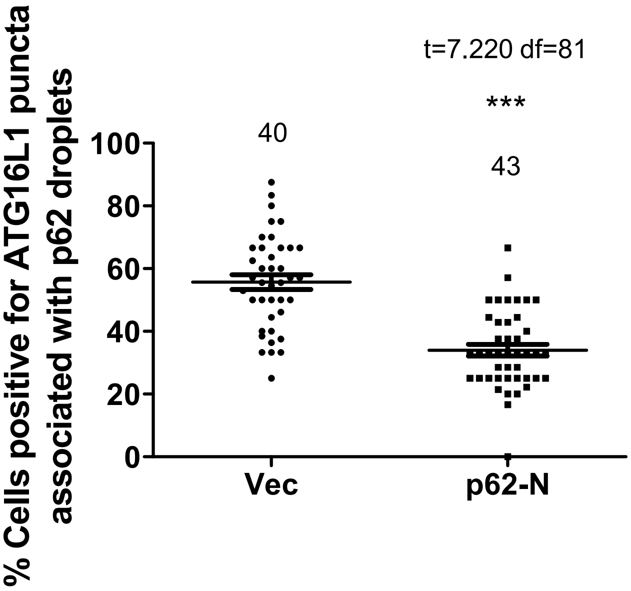

Figure S7. Caspase-6-mediated N-terminal p62 cleavage fragment reduced ATG16L1-positive puncta formation. HeLa cells transfected with mCherry empty vector or mCherry-p62-N were stained with anti-ATG16L1 and anti-p62 (C-terminus) antibody for endogenous ATG16L1 and p62 proteins. Note that anti-p62 C-terminus antibody does not react with mCh-p62-N. Confocal images were acquired (as shown in Fig. 7B). The percentage of cells with ATG16L1 puncta-associated with p62 droplets was assessed. Data are shown as mean±sem. Statistical analysis was performed with unpaired/two-tailed T-test. ****: $P<0.0001$. The n number indicated for each condition represents independent scorings from 3 independently plated wells.

Figure S8

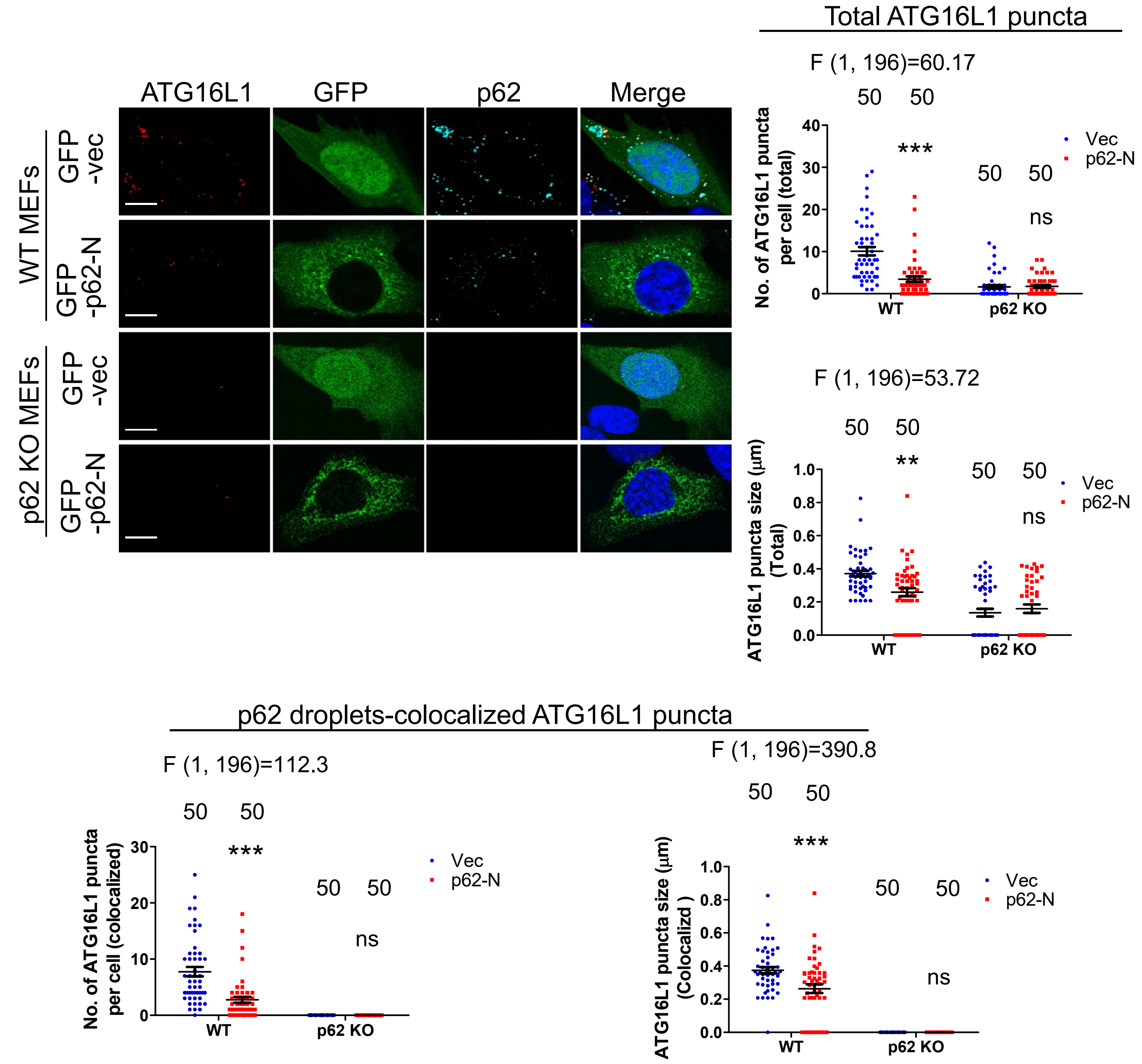

Figure S8. The effect of caspase-6-mediated N-terminal p62 cleavage fragment on ATG16L1 puncta formation was p62-dependent. The effect of caspase-6-mediated N-terminal p62 cleavage fragment on ATG16L1-positive puncta formation in WT or p62 KO MEFs. WT or p62 KO MEFs transfected with empty vector or GFP-p62-N were stained with anti-ATG16L1 and anti-p62 (C-terminus) antibody for endogenous ATG16L1 and p62 proteins. Note that anti-p62 C-terminus antibody does not react with GFP-p62-N. Confocal images were acquired. Bar: 10 μm. Total ATG16L1 puncta in each cell or p62 droplets-associated ATG16L1 puncta in each cell were quantified. The diameter and the count of puncta in each cell were assessed by ImageJ. For the size of ATG16L1 puncta, each point in the plot represents the average size of ATG16L1 puncta in each cell. n=50 cells from 3 independently plated wells. Data are shown as mean±sem. Statistical analysis was performed by Two-way ANOVA with Bonferroni post-tests. The F/degree of freedom/post-hoc p values are indicated in each plot. **: P<0.01; ***: P<0.0001.

Figure S9

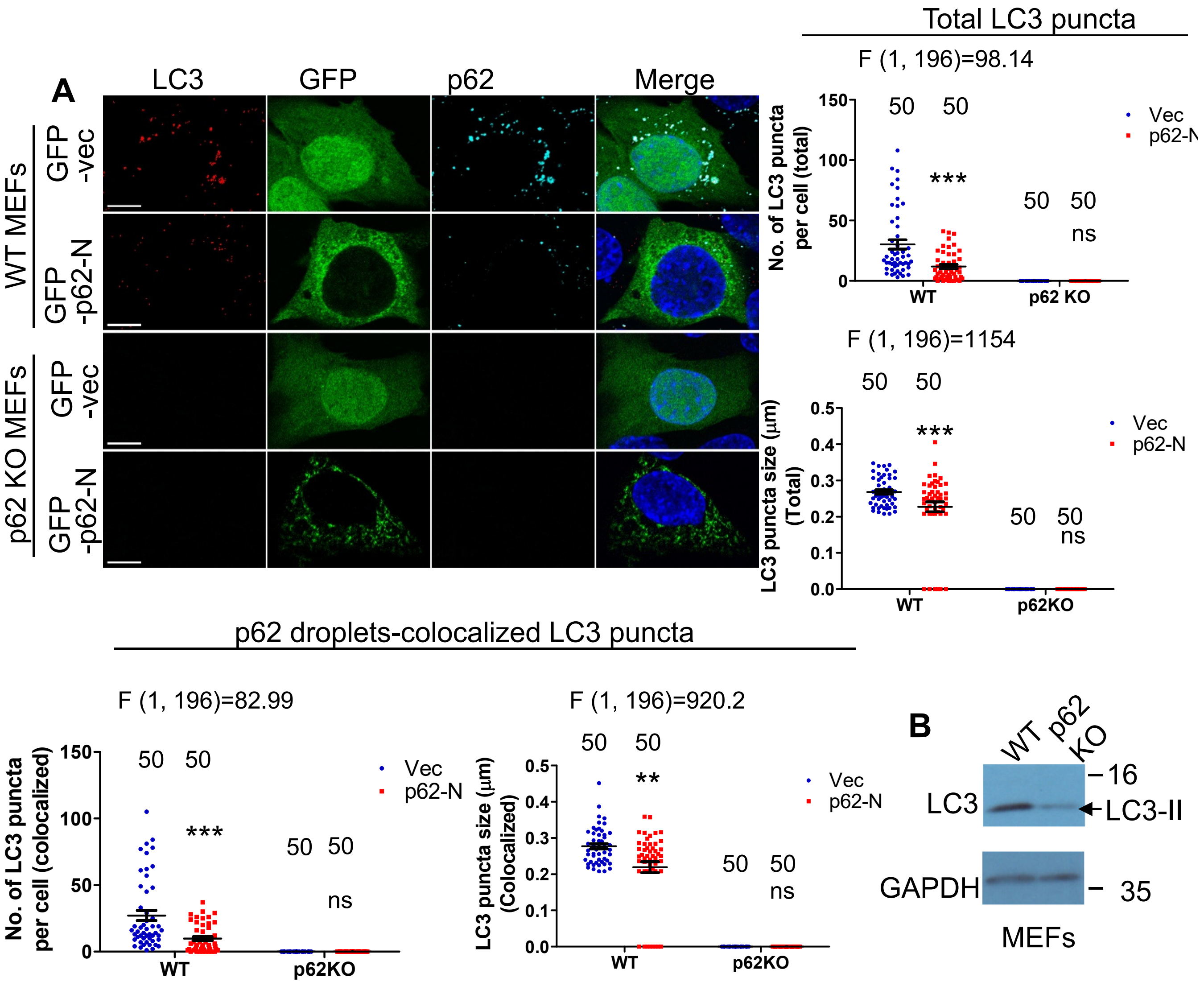

Figure S9. The effect of caspase-6-mediated N-terminal p62 cleavage fragment on LC3-II levels was p62-dependent. (A) The effect of caspase-6-mediated N-terminal p62 cleavage fragment on LC3-positive puncta formation in WT or p62 KO MEFs. WT or p62 KO MEFs transfected with empty vector or GFP-p62-N were stained with anti-LC3 and p62 (C-terminus) antibody for endogenous LC3 and p62 proteins. Note that anti-p62 C-terminus antibody does not react with GFP-p62-N. Confocal images were acquired. Bar: 10 µm. Total LC3 puncta in each cell or p62 droplets-associated LC3 puncta in each cell were quantified. The diameter and the count of puncta in each cell were assessed by ImageJ. For the size of LC3 puncta, each point in the plot represents the average size of LC3 puncta in each cell. n=50 cells from 3 independently plated wells. Data are shown as mean±sem. Statistical analysis was performed by Two-way ANOVA with Bonferroni post-tests. The F/degree of freedom/post-hoc p values are indicated in each plot. *: P<0.05; ***: P<0.0001. Note that in p62 KO MEFs, LC3 vesicle formation dropped at a level lower than the detection threshold of the monoclonal LC3 antibody used in the study. **(B)** The lysates of WT or p62 KO MEFs were subjected to SDS-PAGE and immunoblot with anti-LC3 and GAPDH antibody, successively.

Figure S10

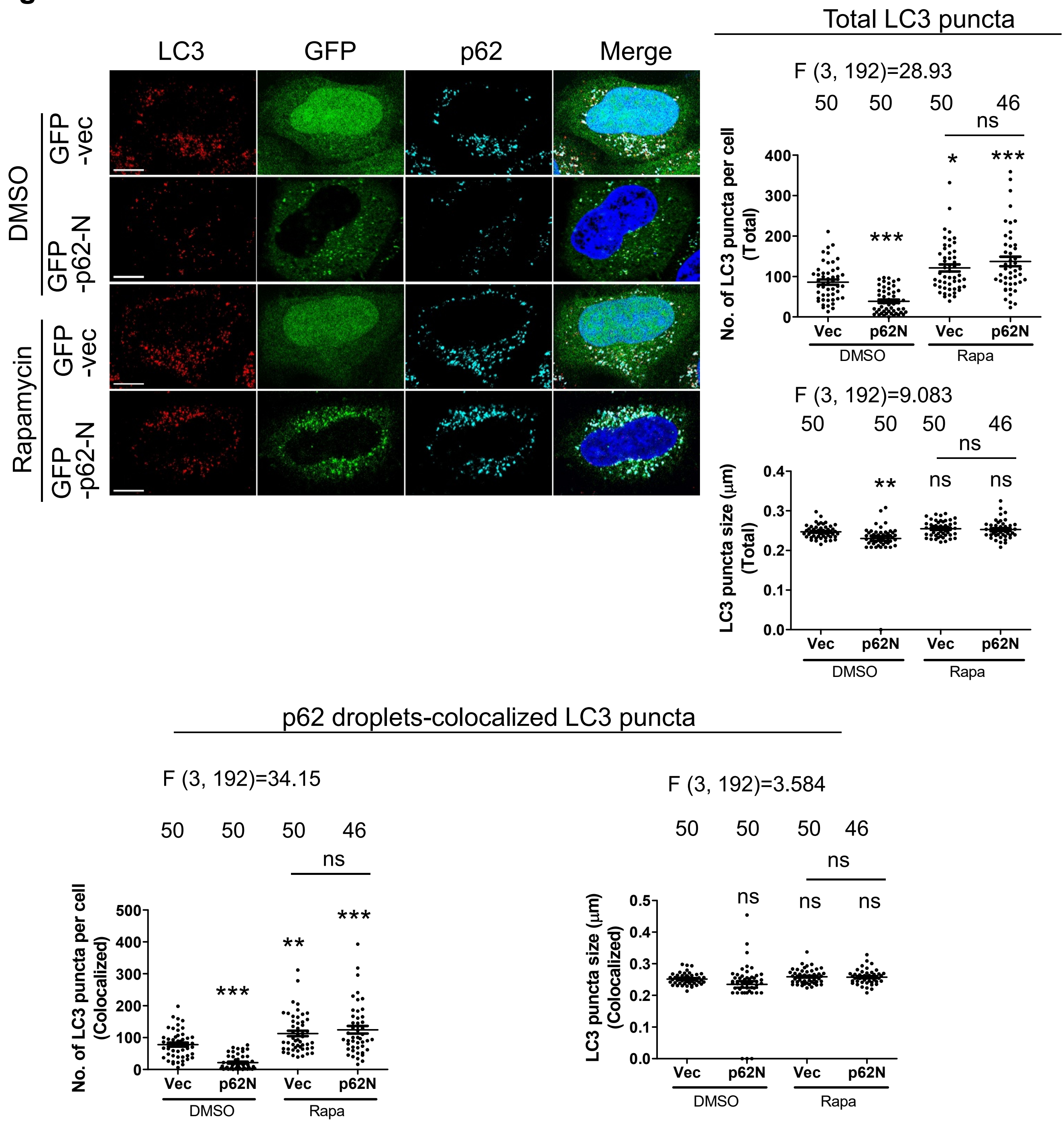

Figure S10. Rapamycin treatment abolished the dominant-negative effect of p62-N on LC3 vesicle formation. HeLa cells were transfected with empty vector or GFP-p62-N. After 3 hours, the transfected cells were treated with vehicle (DMSO) or Rapamycin (2 μM) for 16 hours, as indicated. The cells were stained with anti-LC3 and p62 (C-terminus) antibody for endogenous LC3 and p62 proteins. Bar: 10 μm. Total LC3 puncta in each cell or p62 droplets-associated LC3 puncta in each cell were quantified. The diameter and the count of puncta in each cell were assessed by ImageJ. For the size of LC3 puncta, each point in the plot represents the average size of LC3 puncta in each cell. n= the number of cells from 3 independently plated wells. Data are shown as mean±sem. Statistical analysis was performed by One-way ANOVA with Bonferroni's multiple comparison test. The F/degree of freedom/post-hoc p values are indicated in each plot. ns: not significant; *: $P<0.05$; **: $P<0.01$; ***: $P<0.0001$.

Figure S11

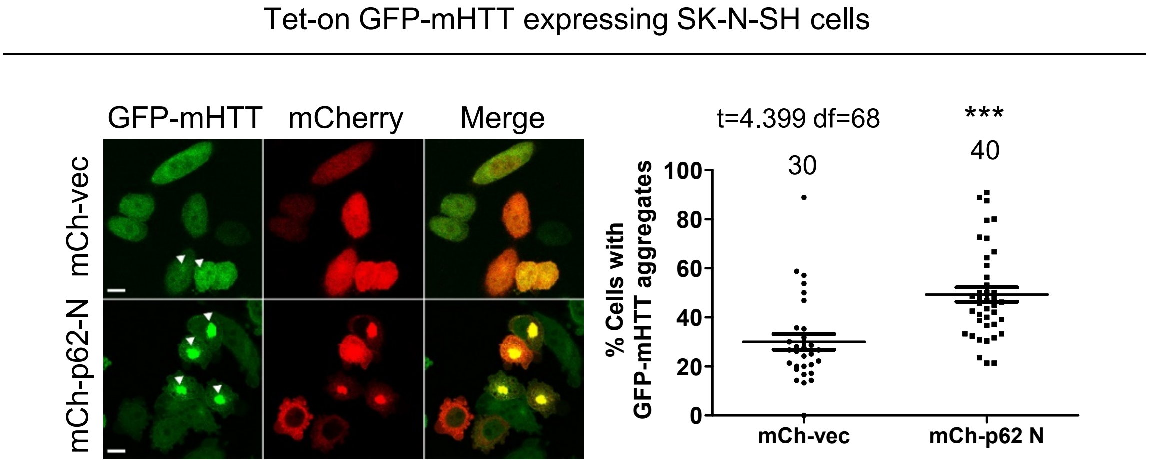

Figure S11. p62-N fragment promoted mHTT aggregation in neuronal cells. SK-N-SH neuroblastoma cells with stable GFP-mHTT Tet-on expression were transfected with mCherry vector or mCherry-p62-N. Confocal images were acquired. Arrows indicate cells with mHTT aggregates. Scale bar: 10 μm. Data are shown as mean±sem. Statistical analysis was performed with unpaired/two-tailed T-test. ****: $P<0.0001$. The n number indicated for each condition represents independent scorings from 3 independently plated wells.

Figure S12

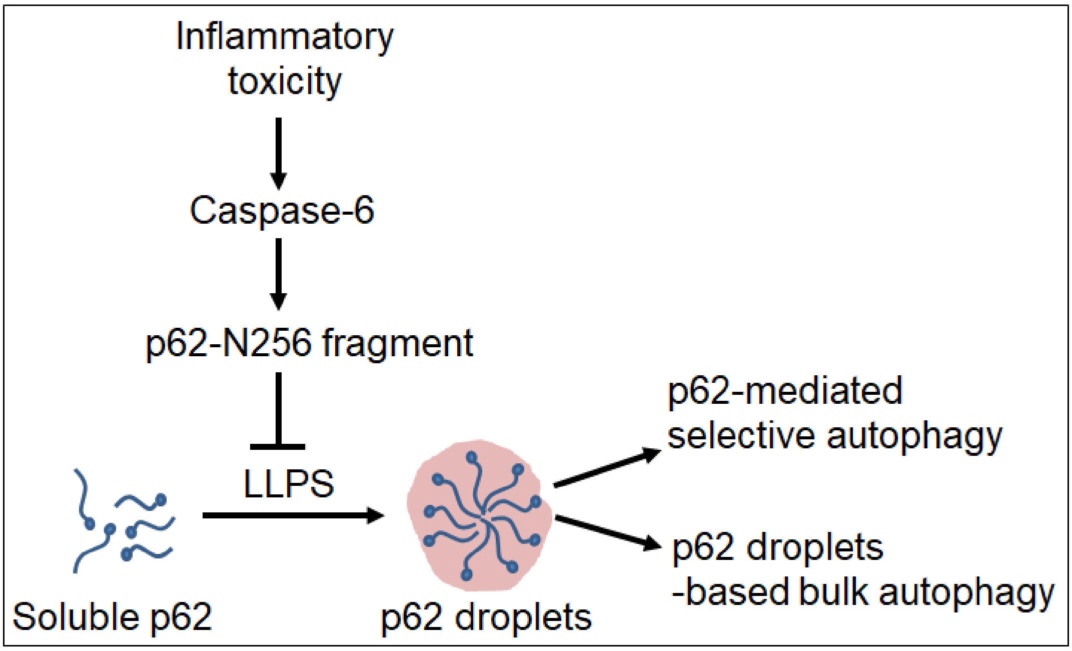

Figure S12. The working model of caspase-6-mediated p62 cleavage in p62 droplets-based autophagy. Inflammatory toxicity induces caspase-6-mediated p62 cleavage at D256, leading to dominant-negative regulation of p62 droplet formation and resulting in attenuation of p62 droplets-based autophagy (p62-mediated selective autophagy and p62-droplets-based bulk autophagy).
